# Metaproteogenomics Reveals Taxonomic and Functional Changes between Cecal and Fecal Microbiota in Mouse

**DOI:** 10.3389/fmicb.2017.00391

**Published:** 2017-03-14

**Authors:** Alessandro Tanca, Valeria Manghina, Cristina Fraumene, Antonio Palomba, Marcello Abbondio, Massimo Deligios, Michael Silverman, Sergio Uzzau

**Affiliations:** ^1^Porto Conte Ricerche, Science and Technology Park of SardiniaAlghero, Italy; ^2^Department of Biomedical Sciences, University of SassariSassari, Italy; ^3^Division of Immunology, Department of Microbiology and Immunobiology, Harvard Medical SchoolBoston, MA, USA; ^4^Division of Infectious Diseases, Department of Pediatrics, Boston Children’s HospitalBoston, MA, USA

**Keywords:** gut microbiome, metabolic pathways, metagenomics, metaproteomics, microbial community, systems microbiology

## Abstract

Previous studies on mouse models report that cecal and fecal microbial communities may differ in the taxonomic structure, but little is known about their respective functional activities. Here, we employed a metaproteogenomic approach, including 16S rRNA gene sequencing, shotgun metagenomics and shotgun metaproteomics, to analyze the microbiota of paired mouse cecal contents (CCs) and feces, with the aim of identifying changes in taxon-specific functions. As a result, Gram-positive anaerobes were observed as considerably higher in CCs, while several key enzymes, involved in oxalate degradation, glutamate/glutamine metabolism, and redox homeostasis, and most actively expressed by Bacteroidetes, were clearly more represented in feces. On the whole, taxon and function abundance appeared to vary consistently with environmental changes expected to occur throughout the transit from the cecum to outside the intestine, especially when considering metaproteomic data. The results of this study indicate that functional and metabolic differences exist between CC and stool samples, paving the way to further metaproteogenomic investigations aimed at elucidating the functional dynamics of the intestinal microbiota.

## Introduction

Compelling evidence has emerged in the last years supporting the gut microbiota as a key factor in mammalian physiology and disease (Marchesi et al., 2016). Mouse models have been increasingly employed to investigate the role and functions of intestinal microbial communities (Laukens et al., 2016). Mouse and human gastrointestinal tracts share many anatomical and functional features, although mouse cecum is relatively larger and able to ferment indigestible food components, while the human cecum is smaller and vestigial (Nguyen et al., 2015). For both human and mouse studies, an essential question regards what kind of sample ought to be collected for achieving the best information on structure and functions of the gut microbiota. In human studies, fecal samples are typically used as a proxy for the gut microbiota, as they are easily accessible; conversely, the collection of luminal or mucosa-associated material directly from the intestine is often unfeasible for ethical and/or practical reasons. When using mouse models, stool is always preferred as sample in time-course studies, because it can be collected from the same mouse throughout the entire duration of the experiment, while cecal samples are collected *post mortem*, often together with other organs to be analyzed.

The main question that comes up is whether, and to what extent, mouse microbiotas from cecal contents (CCs) and feces are comparable in terms of composition and, mostly, functional activity. In fact, many physicochemical conditions (pH, redox potential, oxygen, and salts concentration) as well as biochemical interactions (with molecules released by the intestinal epithelium, immune cells, and other microorganisms), that are expected to shape the microbiota, change along the different regions of the gastrointestinal tract (Haange et al., 2012) and are noticeably different in feces. A few studies analyzed the taxonomic composition of the murine intestinal microbiota at different sampling sites (Pang et al., 2012; Gu et al., 2013; Weldon et al., 2015), but no systematic investigations have been conducted to date. Even more importantly, these surveys were based on denaturation gradient gel electrophoresis or 16S rRNA gene sequencing, with no information about genetic potential and functional activities of the microbiota, which can be achieved using shotgun metagenomics and metaproteomics, respectively.

Here, we compared the microbiota of paired mouse CCs and feces (F) in order to investigate the structural and functional differences between the two microbial communities. To this aim, we chose to employ a metaproteogenomic approach, enabling the elucidation of the active response of the microbiota to the environmental perturbations through the identification of its actually expressed proteins.

## Materials and Methods

### Mice and Sample Collection

Samples were collected from three 10-week-old female NOD mice bred at the specific pathogen free facility in the New Research Building at Harvard Medical School. Mice were provided a standard chow diet *ad libitum* (PicoLab Mouse Diet 20, #5058, LabDiet, St. Louis, MO, USA). Fresh fecal pellets (approximately 100 mg for each mouse) were collected into sterile tubes under a laminar flow hood, immediately placed on dry ice and then stored at -80°C until processing. To obtain the cecal luminal content, mice were sacrificed at the same time point of fecal collection. Ceca were immediately collected, opened longitudinally, and vigorously shaken in 5 ml of sterile PBS to release their contents. The cecal tissue was removed and the contents were then centrifuged at 10,000 x *g* for 10 min. The supernatants were discarded, while the pellets (approximately 50 mg each) were flash frozen in liquid nitrogen and stored at -80°C until use. This study was carried out in accordance with the recommendations of the Institutional Animal Care and Use Committee of Harvard Medical School, and the experimental protocol was approved by the same Committee. At the time of the analyses, fecal samples and CCs were thawed at 4°C, and from each of them two portions were collected for DNA and protein extraction, respectively.

### DNA Extraction and 16S rRNA Gene Analysis

DNA extraction was undertaken using the QIAamp DNA Stool Mini Kit (Qiagen, Hilden, Germany), according to the manufacturer’s protocol. Amplification of the entire 16S-rRNA genes was performed using the universal primers 27F-1492R (AGAGTTTGATYMTGGCTCAG and TACGGYTACCTTGTTACGACTT, respectively) and the recombinant Taq DNA Polymerase from Invitrogen (Thermo Scientific, San Jose, CA, USA). PCR cycling conditions were as follows: 2 min at 94°C; 28 cycles of 30 s at 94°C, 30 s at 55°C, 2 min at 68°C; finally, 7 min at 72°C. PCR products were confirmed on 2% agarose gel (Sigma Aldrich, St. Louis, MO, USA). The 16S rRNA gene amplification reaction was performed in duplicate, then the two amplification products were pooled together, cleaned up using AMPure XP (Beckman Coulter, Brea, CA, USA) magnetic beads and quantified with the Qubit HS assay using the Qubit fluorimeter 2.0 (Life Technologies, Grand Island, NY, USA).

Libraries were constructed according to the Nextera XT kit (Illumina, San Diego, CA, USA). The average insert size was around 500 bps. Sequence-ready libraries were normalized to ensure equal library representation in the pooled samples. DNA sequencing was performed with the Illumina HiScanSQ sequencer, using the paired-end method and 93 cycles of sequencing.

The Illumina demultiplexed paired-reads were trimmed for the first 20 bps using FASTX and the sequences with Nextera adapter contamination were identified using the UniVec database^1^ and removed. Paired reads were merged using the script join_paired_ends.py inside the QIIME package v.1.9.0 (Caporaso et al., 2010) with a minimum overlap of eight base pairs. OTU generation was done using a QIIME pipeline based on USEARCH’s OTU clustering recommendations^2^ using the closed-reference OTU picking to allow clustering of shotgun 16S sequences. Reads were clustered at 97% identity using UCLUST to produce OTUs (Edgar, 2010). Taxonomy assignment of resulting OTUs was performed using the Greengenes 13_8 database (DeSantis et al., 2006). With taxonomic lineages in hand, OTU tables were computed using QIIME (Caporaso et al., 2010; Kuczynski et al., 2010).

### Metagenome Analysis

Libraries were constructed according to the Nextera XT kit and sequenced with the HiScanSQ sequencer (both from Illumina), using the paired-end method and 93 cycles of sequencing.

Read processing (merging of paired reads and quality filtering) was carried out using tools from the USEARCH suite v.8.1.1861 (Edgar, 2010; Edgar and Flyvbjerg, 2015), using the parameters described elsewhere (Tanca et al., 2016).

Taxonomic annotation was performed using MEGAN v.5.11.3 (Huson and Mitra, 2012). Read sequences were preliminary subjected to DIAMOND (v.0.7.1) search against the NCBI-nr DB (2016/03 update), using the blastx command with default parameters (Buchfink et al., 2015). Then, a lowest common ancestor (LCA) classification was performed on DIAMOND results using MEGAN with default parameters.

Functional annotation was accomplished by DIAMOND blastx search (e-value threshold 10^-5^) against bacterial sequences from the UniProt/Swiss-Prot database (release 2015_12) and subsequent retrieval of protein family, KEGG orthologous group and pathway information associated with each UniProt/Swiss-Prot accession number (UniProt Consortium, 2015).

The metagenomic sequence data were deposited in the European Nucleotide Archive under the project accession number PRJEB15341.

### Protein Sample Preparation

Samples were resuspended by vortexing in SDS-based extraction buffer and then heated and subjected to a combination of bead-beating and freeze-thawing steps as detailed elsewhere (Tanca et al., 2014).

Protein extracts were subjected to on-filter reduction, alkylation, and trypsin digestion according to the filter-aided sample preparation (FASP) protocol (Wisniewski et al., 2009), with slight modifications detailed elsewhere (Tanca et al., 2013).

### Metaproteome Analysis

LC-MS/MS analysis was carried out using an LTQ-Orbitrap Velos mass spectrometer (Thermo Scientific) interfaced with an UltiMate 3000 RSLCnano LC system (Thermo Scientific). The single-run 1D LC peptide separation was performed as previously described (Tanca et al., 2014), loading 4 μg of peptide mixture per each sample and applying a 485 min separation gradient. The mass spectrometer was set up in a data dependent MS/MS mode, with Higher Energy Collision Dissociation as the fragmentation method, as detailed elsewhere (Tanca et al., 2013).

Peptide identification was performed using the Proteome Discoverer informatic platform (version 1.4; Thermo Scientific), with Sequest-HT as search engine and Percolator for peptide validation (FDR < 1%). Search parameters were set as described previously (Tanca et al., 2015).

Parallel searches were performed using two different sequence databases, and results from the two searches for each sample were merged. The first database was composed of the metagenomic sequences obtained in this study, both as raw reads and assembled contigs (2,158,809 sequences). Paired reads were merged as described above for 16S rRNA gene analysis. The output sequences were filtered (with a fastq_truncqual option = 15) and clustered at 100% using USEARCH v.5.2.236 (Edgar, 2010). Read assembly into contigs was carried out using Velvet v.1.2.10 (Zerbino and Birney, 2008), by setting 61 as k-mer length, 200 as insert length, and 300 as minimum contig length. Open reading frames were found from both reads and contigs using FragGeneScan v.1.19, with the training for Illumina sequencing reads with about 0.5% error rate (Rho et al., 2010). The second database was a selection of all bacterial, archaeal, fungal and gut microbiota sequences (79,203,800 sequences in total) from the 2015_02 release of the UniProtKB database.

The mass spectrometry proteomics data have been deposited to the ProteomeXchange Consortium via the PRIDE (Vizcaino et al., 2016) partner repository with the dataset identifier PXD004911.

The Normalized Spectral Abundance Factor (NSAF) was calculated in order to estimate protein abundance (Zybailov et al., 2006). Specifically, a spectral abundance factor (SAF) was obtained by dividing the number of spectral counts of a protein by its number of amino acids (Zhang et al., 2010); then, NSAF values were obtained by dividing the SAF values by the SAF sum for all proteins identified in each sample. NSAF values were finally multiplied by a scale factor corresponding to the average number of spectral counts identified per sample (in order to deal with integers).

Taxonomic and functional assignments were performed as described above for metagenome sequences, except using the DIAMOND blastp command instead of blastx. Moreover, peptides from sequences which could not be annotated by MEGAN were iteratively subjected to LCA taxonomic classification using the ‘Metaproteome analysis’ module of the Unipept web application (Mesuere et al., 2015).

### Statistical Analysis and Graph Generation

Differential analysis of 16S-MG, S-MG and MP data (adjusted based on the total number of counts per sample) was performed using an established paired sample test for count data based on an inverted beta binomial (ibb) model (Pham and Jimenez, 2012). Meta-omic count data were assumed to be modeled by a beta binomial distribution based on previous studies performed on count data obtained through discovery mass spectrometry proteomics (Ramus et al., 2016) and nucleic acid sequencing techniques (Smith and Birtwistle, 2016). The *p*-value list provided by the ibb test was subsequently subjected to a multiple testing adjustment based on a sequential goodness of fit (SGoF) metatest (Carvajal-Rodriguez et al., 2009) using the SGoF+ software (v.3.8) with default parameters (Carvajal-Rodriguez and de Una-Alvarez, 2011). This metatest has been successfully applied to large RNA-seq and proteomic datasets (Shi et al., 2012; Mortstedt et al., 2015), and was applied in this study in view of its suitability for small sample size and ability to maintain a high statistical power when increasing the number of test. An adjusted *p*-value < 0.05 was considered as the threshold for statistical significance of differential results.

Fold-change was calculated in a paired sample fashion (mean of cecum/feces ratios calculated for each individual mouse), using a correction factor (CF = 2) to eliminate discontinuity due to missing values; fold-change values that were less than 1 were replaced by the negative of their inverse. Furthermore, features with missing values in the most abundant group were filtered out from those considered as differentially abundant.

Shannon’s index for alpha diversity estimation was calculated according to established methods (Hill et al., 2003). ClustVis^3^ was employed to generate PCA plots (Metsalu and Vilo, 2015). Cladograms were generated using GraPhlAn (Asnicar et al., 2015) and edited using Inkscape^4^. Venn diagrams were plotted using Venn Diagram Plotter^5^.

## Results

### General Metaproteogenomic Metrics of Mouse Cecal and Fecal Microbiota

The number of reads sequenced per sample, and taxonomically annotated at least at the phylum level, ranged from 97,929 to 305,577 for 16S-MG (mean 177,994), and from 316,074 to 2,119,077 for S-MG (mean 1,028,635). Based on MP data, the number of peptide-spectrum matches identified per sample, and taxonomically assigned at least at the phylum level, ranged from 14,786 to 16,901 (mean 16,264). The number of OTUs detected by 16S-MG ranged from 2,421 to 7,390 (mean 4,865). The levels of overlap among OTUs, genera and functions identified with the three omic approaches in CC and F samples are illustrated in the Venn diagrams of Supplementary Figures S1–S7.

We first compared alpha-diversity in CC and F samples (Supplementary Figure S8), both based on taxonomic and functional data. On the whole, no univocal and significant differences could be observed between CC and F (probably due to the small sample size and a considerable inter-individual variability), apart from a significantly higher taxonomic diversity in CC according to MP data. Principal component analysis (PCA) of taxonomic and functional data obtained with the three meta-omic approaches (Supplementary Figure S9) also suggested a considerable impact of inter-individual variability, although clustering of CC and F samples could be observed on the second component when considering taxon and function abundances based on MP data.

### Cecal and Fecal Microbiota Exhibit Different Taxonomic Structures

**FIGURE 1:**
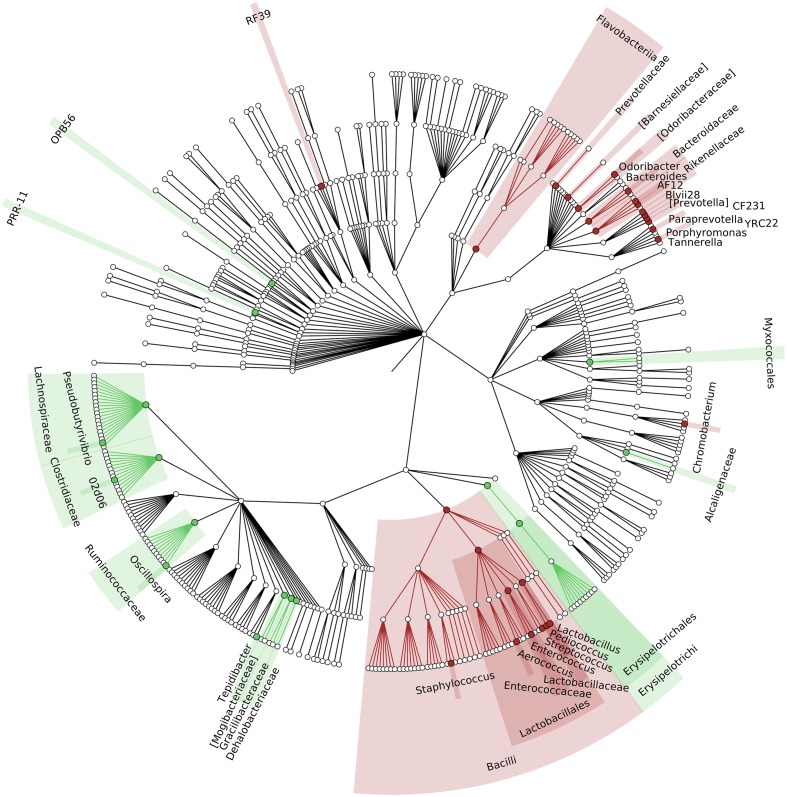
**Cladogram illustrating the distribution of taxa with differential abundance between cecal contents (CCs) and feces, according to 16S-MG data**. Each dot represents an identified taxon, with taxa higher in CCs colored in green, and those higher in feces in brown.

**FIGURE 2:**
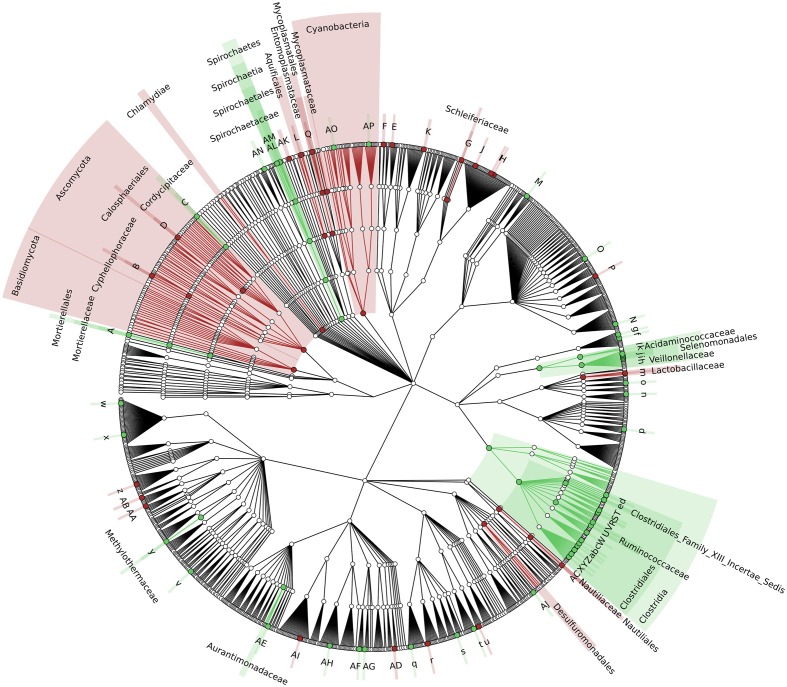
**Cladogram illustrating the distribution of taxa with differential abundance between CCs and feces, according to S-MG data**. Each dot represents an identified taxon, with taxa higher in CCs colored in green, and those higher in feces in brown. Abbreviations: A, *Mortierella*; a, *Lachnoclostridium*; AA, *Zymobacter*; AB, *Marinomonas*; AC, *Lebetimonas*; AD, *Candidatus Pelagibacter*; AE, *Microvirga*; AF, *Wolbachia*; AG, *Candidatus Paracaedibacter*; AH, *Magnetospira*; AI, *Haematobacter*; AJ, *Chondromyces*; AK, *Thermoanaerobaculum*; AL, *Treponema*; AM, *Brachyspira*; AN, *Cloacibacillus*; AO, *Mastigocoleus*; AP, *Acaryochloris*; B, *Cyphellophora*; b, *Youngiibacter*; C, *Beauveria*; c, *Clostridium*; d, *Oscillibacter*; D, *Togninia*; e, *Anaerovorax*; E, *Segetibacter*; f, *Dielma*; F, *Phaeodactylibacter*; g, *Faecalitalea*; G, *Schleiferia*; H, *Algibacter*; h, *Mitsuokella*; I, *Psychroserpens*; i, *Selenomonas*; J, *Cellulophaga*; j, *Sporomusa*; K, *Flexibacter*; k, *Megasphaera*; l, *Megamonas*; L, *Persephonella*; m, *Lactobacillus*; M, *Olsenella*; n, *Alkalibacterium*; N, *Pseudonocardia*; o, *Eremococcus*; O, *Microbispora*; P, *Dactylosporangium*; p, *Saccharibacillus*; q, *Acidovorax*; Q, *Mycoplasma*; r, *Azohydromonas*; R, *Flavonifractor*; S, *Intestinimonas*; s, *Thiomonas*; t, *Chitiniphilus*; T, *Epulopiscium*; u, *Conchiformibius*; U, *Ruminococcus*; v, *Anaerobiospirillum*; V, *Anaerotruncus*; w, *Budvicia*; W, *Dehalobacter*; X, *Lachnospira*; x, *Raoultella*; Y, *Butyrivibrio*; y, *Methylohalobius*; z, *Lysobacter*; Z, *Shuttleworthia*.

**FIGURE 3:**
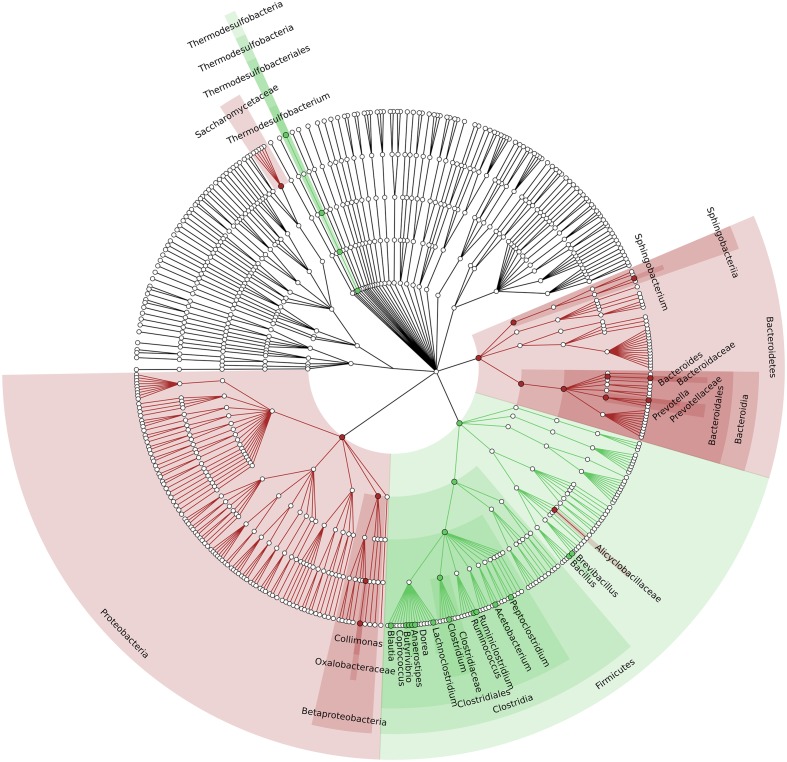
**Cladogram illustrating the distribution of taxa with differential abundance between CCs and feces, according to MP data**. Each dot represents an identified taxon, with taxa higher in CCs colored in green, and those higher in feces in brown.

To identify which members of the microbiota changed in relative abundance when the intestinal contents moved from the mouse cecum to the external environment, we pairwise compared taxa abundances measured in CC and F based on 16S-MG, S-MG and MP analysis. Cladograms in **Figures 1**–**3** illustrate in a hierarchical fashion 44, 101, and 36 differentially abundant taxa detected according to 16S-MG, S-MG, and MP data, respectively (the overlap among differential genera according to the three different approaches is illustrated in the Venn diagram of Supplementary Figure S10). As a main result, clostridia (Gram-positive anaerobes, including many members of Lachnospiraceae, Ruminococcaceae, and Clostridiaceae) were found to be significantly more represented in CC compared to F, according to all approaches. On the other hand, MG data revealed that some Gram-positive aerobes (e.g., those belonging to the family Lactobacillaceae) were significantly more abundant in F compared to CC, as well as several taxa from Bacteroidetes (including *Bacteroides* and *Prevotella*, particularly according to 16S-MG and MP).

### Specific Phylum-Related Functions Differ between Cecal and Fecal Microbiota

We then focused on the functions encoded (S-MG) and expressed (MP) by the CC and F microbiota. Specifically, we combined functional (retrieved from the KEGG Orthology database) and taxonomic information (phylum) assigned to each sequence, in order to answer to the basic questions ‘who is able to do what’ (S-MG results) and ‘who is actually doing what’ (MP results).

According to S-MG results, we found that 495 function-phylum combinations were significantly more represented in CC than in F, while 186 were differential in the opposite direction (Supplementary Table S1). More interestingly, and consistently with taxonomic data, over 97% of functions higher in CC belonged to Firmicutes, while 58, 17, and 12% of those higher in F had been assigned to Bacteroidetes, Proteobacteria, and Actinobacteria, respectively. On the other hand (**Table 1** and Supplementary Table S2), 49 function-phylum combinations resulted as more abundant in CC according to MP data, while 34 were higher in F. Again, 94% of functions more represented in CC belonged to Firmicutes, whereas all but one of those higher in F were from Bacteroidetes. The overlap between the differential function-phylum combinations detected with S-MG and MP is provided in the Venn diagram of Supplementary Figure S11.

**Table 1:** Functional-taxonomic features with significantly differential abundance between cecal and fecal metaproteome.

Function	Phylum	Adjusted *p*-value	Mean CC/F fold-change (bbbSEM)
Methyl-accepting chemotaxis protein	Firmicutes	2.7 × 10^-33^	8.36 ±5.41
Iron(III) transport system substrate-binding protein	Firmicutes	4.3 × 10^-53^	6.92 ±4.80
Phosphoribosylaminoimidazole-succinocarboxamide synthase	Firmicutes	4.4 × 10^-37^	5.24 ±2.75
Acyl carrier protein	Firmicutes	3.2 × 10^-60^	5.06 ±1.70
ATP-dependent Clp protease, protease subunit	Firmicutes	5.2 × 10^-55^	4.72 ±2.01
Acetate CoA-transferase	Firmicutes	7.7 × 10^-52^	4.44 ±1.82
Aspartyl-tRNA synthetase	Firmicutes	4.0 × 10^-21^	4.34 ±2.59
3D-(3,5/4)-Trihydroxycyclohexane-1,2-dione acylhydrolase	Firmicutes	2.2 × 10^-19^	4.31 ±2.23
Phosphoglucomutase	Firmicutes	9.4 × 10^-39^	4.27 ±1.05
Polyribonucleotide nucleotidyltransferase	Firmicutes	6.9 × 10^-61^	4.27 ±0.29

Large subunit ribosomal protein L13	Bacteroidetes	2.4 × 10^-10^	-5.16 ±3.06
Superoxide dismutase, Fe–Mn family	Bacteroidetes	9.7 × 10^-49^	-5.49 ±1.93
Peroxiredoxin (alkyl hydroperoxide reductase subunit C)	Bacteroidetes	2.3 × 10^-34^	-5.62 ±2.87
Flagellin	Proteobacteria	7.6 × 10^-24^	-6.17 ±4.44
Pullulanase	Bacteroidetes	1.4 × 10^-63^	-9.25 ±3.70
Molecular chaperone DnaK	Bacteroidetes	6.0 × 10^-15^	-9.91 ±4.81
Glutaminase	Bacteroidetes	3.0 × 10^-64^	-11.39 ±2.27
Oxalyl-CoA decarboxylase	Bacteroidetes	2.7 × 10^-66^	-13.72 ±4.33
Glutamate decarboxylase	Bacteroidetes	1.3 × 10ccc	-17.08 ±6.43
Formyl-CoA transferase	Bacteroidetes	6.3 × 10ccc	-22.09 ±4.62

Since most of the observed differences seemed to be directly dependent on changes in Firmicutes-to-Bacteroidetes ratio, we decided to further normalize the abundance of Firmicutes and Bacteroidetes functions based on the total amount of each specific phylum in a given sample, with the aim of finding those functions changing independently of the ‘structural’ (taxonomic) modification of the microbiota. When considering S-MG data for Firmicutes, we found 45 gene functions higher in CC (including enzymes involved in sulfur metabolism and components of bacterial flagella) and 50 gene functions higher in F (Supplementary Table S3), while MP data showed 10 protein functions as more abundant in CC versus 3 more abundant in F (**Table 2**). ABC transporters (with an iron transport system protein changing >6-fold in expression), proteases/peptidases, and enzymes involved in purine metabolism were among specific Firmicutes functions ‘active’ in CC and ‘silenced’ in F. When focusing on Bacteroidetes, 4 and 17 gene (mainly catalytic) functions were detected as more abundant in CC and F, respectively (Supplementary Table S4); furthermore, based on MP data, 3 and 12 protein functions were enriched and depleted in CC compared to F samples, respectively (**Table 3**). Several key enzymes involved in oxalate degradation, glutamate/glutamine metabolism, and redox homeostasis were identified among those expressed by Bacteroidetes with increased relative abundance in F compared to CC, often with remarkable fold-changes (up to over 10).

**Table 2:** Firmicutes functions with significantly differential normalized abundance between cecal and fecal metaproteome.

Function	Adjusted	Mean CC/F
	*p*-value	fold-change (±SEM)
Iron(III) transport system substrate-binding protein	4.7 × 10^-6^	6.07 ±4.47
Acyl carrier protein	2.9 × 10^-8^	4.28 ±1.48
Phosphoribosylaminoimidazole-succinocarboxamide synthase	1.3 × 10^-3^	4.27 ±2.08
ATP-dependent Clp protease, protease subunit	1.7 × 10^-7^	4.11 ±1.81
Acetate CoA-transferase	6.8 × 10^-4^	3.72 ±1.89
Polyribonucleotide nucleotidyltransferase	1.0 × 10^-5^	2.67 ±0.33
Phosphoglucomutase	2.3 × 10^-3^	2.41 ±0.46
Phenylalanyl-tRNA synthetase beta chain	4.0 × 10^-7^	2.33 ±0.17
Presequence protease	2.1 × 10^-6^	2.17 ±0.33
Spermidine/putrescine transport system ATP-binding protein	4.5 × 10^-5^	2.00 ±0.29

Peptide/nickel transport system substrate-binding protein	1.2 × 10^-2^	-1.46 ±0.13
Glyceraldehyde 3-phosphate dehydrogenase	9.3 × 10^-7^	-1.46 ±0.08
2,3-Bisphosphoglycerate-dependent phosphoglycerate mutase	9.2 × 10^-5^	-2.90 ±0.56

**Table 3:** Bacteroidetes functions with significantly differential normalized abundance between cecal and fecal metaproteome.

Function	Adjusted	Mean CC/F f
	*p*-value	old-change (±SEM)
Large subunit ribosomal protein L11	1.1 × 10^-2^	4.84 ±3.08
F-type H^+^-transporting ATPase subunit beta	3.8 × 10^-2^	3.04 ±1.20
L-Rhamnose isomerase	5.7 × 10^-4^	1.76 ±0.13

Deoxyribose-phosphate aldolase	2.5 × 10^-4^	-1.65 ±0.13
Catalase	2.7 × 10^-3^	-1.67 ±0.17
Prolyl-tRNA synthetase	5.5 × 10^-3^	-1.67 ±0.17
Thioredoxin reductase (NADPH)	2.9 × 10^-7^	-2.33 ±0.44
4-Alpha-glucanotransferase	9.8 × 10^-9^	-2.83 ±1.09
L-Asparaginase	9.6 × 10^-8^	-3.28 ±0.43
Superoxide dismutase, Fe–Mn family	2.1 × 10^-2^	-3.75 ±1.59
Pullulanase	9.0 × 10-10	-6.60 ±2.55
Glutaminase	5.5 × 10^-12^	-8.33 ±1.69
Formyl-CoA transferase	1.4 × 10^-12^	-9.53 ±1.65
Oxalyl-CoA decarboxylase	7.4 × 10^-11^	-10.18 ±4.18
Glutamate decarboxylase	2.0 × 10^-11^	-12.99 ±6.15

### Activity of Metabolic Pathways Changes between Cecal and Fecal Microbiota

To gain insight into the metabolic activity of the microbiota, we aggregated phylum-assigned functional data according to the metabolic pathway to which each sequenced (S-MG) or identified (MP) enzyme could be assigned based on UniProtKB information. As a result (Supplementary Table S5), 110 pathways resulted as differentially represented between CC and F metagenomes, with 97% of those more represented in CC being assigned to Firmicutes, while 44, 26, and 10% of those higher in F being assigned to Bacteroidetes, Proteobacteria, and Actinobacteria, respectively (consistently with general functional data). Of interest, a marked drop could be observed in F in the relative abundance of genes responsible for the degradation of various di- and polysaccharides, as well as in the sulfur and butyrate metabolism, counterbalanced by an increase in genetic potential toward biosynthetic routes due to several non-Firmicutes phyla. When normalizing on the total abundance of Firmicutes in the samples, 19 pathways remained differential (including 3-phenylpropanoate degradation, sulfite reduction, hydrogen sulfide biosynthesis and tetrahydrofolate biosynthesis more represented in CC, and L-tryptophan degradation, lactose degradation, starch degradation and pentose phosphate pathway more represented in F), indicating that some differences were not proportional to the general taxonomic modification of the microbiota between CC and F.

As shown in **Table 4**, 18 pathways were found to be more active in the CC metaproteome (89% from Firmicutes), including tetrahydrofolate interconversion (part of the Wood–Ljungdahl pathway), pentose phosphate pathway, as well as those related to pyruvate and short chain fatty acid metabolism. In addition, starch degradation from Ascomycota (fungi) and Proteobacteria were observed to be clearly higher in CC compared to F. On the other hand, all 14 pathways whose enzymes were more expressed in F belonged to Bacteroidetes; among them, lipid IV(A) biosynthesis, urea degradation, purine nucleoside salvage and oxalate degradation (this latter pathway with a mean fold-change > 20) were confirmed to be significantly differential even upon normalization on the total abundance of Bacteroidetes in the samples.

**Table 4:** Differential pathway-phylum combinations between cecal and fecal metaproteome.

Pathway	Phylum	Adjusted *p*-value	Mean CC/F fold-change (±SEM)
Tetrahydrofolate interconversion	Firmicutes	1.5 × 10^-3^	6.00 ±3.80
IMP biosynthesis via *de novo* pathway	Firmicutes	4.7 × 10^-16^	4.92 ±1.51
Diglucosyl-diacylglycerol biosynthesis	Firmicutes	1.1 × 10^-19^	4.62 ±1.11
Pentose phosphate pathway	Firmicutes	1.0 × 10^-4^	3.86 ±1.57
Starch degradation	Ascomycota	1.4 × 10^-5^	3.83 ±2.13
Pyruvate fermentation	Firmicutes	1.6 × 10^-6^	3.53 ±1.24
1,2-Propanediol degradation	Firmicutes	1.0 × 10^-12^	3.22 ±0.89
Galactarate degradation	Firmicutes	4.4 × 10^-12^	3.18 ±1.06
(R)-Mevalonate biosynthesis	Firmicutes	6.5 × 10^-4^	2.84 ±0.87
Propanoyl-CoA degradation	Firmicutes	4.9 × 10^-6^	2.67 ±0.60
L-Tryptophan degradation via pyruvate pathway	Firmicutes	2.7 × 10^-4^	2.36 ±0.22
L-Isoleucine biosynthesis	Firmicutes	9.4 × 10^-17^	2.23 ±0.32
Glycogen biosynthesis	Firmicutes	4.0 × 10^-9^	2.23 ±0.31
UMP biosynthesis via salvage pathway	Firmicutes	5.2 × 10^-14^	2.17 ±0.67
Starch degradation	Proteobacteria	2.3 × 10^-15^	2.17 ±0.33
L-Arabinose degradation via L-ribulose	Firmicutes	1.1 × 10^-14^	2.02 ±0.25
Glycolysis	Firmicutes	2.4 × 10^-13^	1.98 ±0.28
Butanoate metabolism	Firmicutes	5.5 × 10^-22^	1.87 ±0.06

Glycolysis	Bacteroidetes	2.9 × 10^-10^	-1.79 ±0.22
Tricarboxylic acid cycle	Bacteroidetes	1.8 × 10^-17^	-1.80 ±0.12
Xyloglucan degradation	Bacteroidetes	1.1 × 10^-9^	-2.31 ±0.36
Selenocysteinyl-tRNA(Sec) biosynthesis	Bacteroidetes	1.8 × 10^-11^	-2.44 ±0.29
L-Lysine biosynthesis via DAP pathway	Bacteroidetes	4.9 × 10^-8^	-2.60 ±0.31
Starch degradation	Bacteroidetes	6.3 × 10^-19^	-2.74 ±0.42
2-Dehydro-3-deoxy-D-gluconate degradation	Bacteroidetes	3.4 × 10^-18^	-3.00 ±0.76
L-Isoleucine biosynthesis	Bacteroidetes	1.4 × 10^-8^	-3.04 ±0.69
IMP biosynthesis via *de novo* pathway	Bacteroidetes	5.2 × 10^-7^	-3.20 ±1.20
2-Deoxy-D-ribose 1-phosphate degradation	Bacteroidetes	3.3 × 10^-21^	-3.37 ±0.29
Lipid IV(A) biosynthesis	Bacteroidetes	2.0 × 10^-20^	-4.44 ±1.56
Urea degradation	Bacteroidetes	3.3 × 10^-25^	-5.67 ±1.09
Purine nucleoside salvage	Bacteroidetes	2.2 × 10^-24^	-7.22 ±2.69
Oxalate degradation	Bacteroidetes	4.9 × 10^-26^	-22.15 ±8.10

## Discussion

The main purpose of this study was to identify taxon-specific functions changing between CCs and stool by means of a metaproteogenomic approach. Information about microbial functional traits actually changing in response to stimuli from host, diet, or other environmental factors can be in fact only gathered by functional meta-omics, in view of their intrinsic sensitivity to perturbation (Heintz-Buschart et al., 2016). In particular, metaproteomics is able to measure microbial proteins, which represent key molecules in GM metabolism and host-GM interaction (Xiong et al., 2015). Here, in spite of the small sample size employed, the global analytical strategy allowed the identification of several phylum-specific metabolic pathways differing in activity between these two microbial communities, confirming metaproteogenomics as a promising tool to unveil microbiota functional variations. On the whole, the structure of the fecal microbiota appeared to differ moderately from that of the luminal cecal microbiota, in line with previous reports (Gu et al., 2013), with the large majority of the taxonomic features identified in both CC and F samples, according to all approaches. However, some substantial changes in the functional and metabolic activity could be observed, especially and more effectively as microbial functionalities were evaluated by means of an integrated metaproteogenomic approach, where the use of matched metagenomes as sequence databases significantly improve the protein identification yield (Tanca et al., 2016).

We chose to employ a full-length 16S rRNA amplification due to technical reasons related to the specific sequencer used in the study, as well as in view of preliminary tests in which a slightly higher richness and diversity was measured when amplifying the full-length 16S rRNA sequence rather then the single V4 hypervariable region (data not shown). Although a closed-reference OTU picking was applied, which is specifically designed to allow clustering of shotgun 16S sequences (similar to the randomly distributed 16S rRNA fragments sequenced in this work), it has to be noted that the presence of some reads containing highly conserved sequence portions may have led to a slight decrease in taxonomic resolution, and/or to a minor increase in false positive taxonomic assignments. Moreover, the low level of comparability between 16S and S-MG taxonomic results observed in this study might be likely due to differences in taxonomic classification and update frequency among databases (GreenGenes vs. NCBI).

In this study, fresh fecal pellets were collected within few minutes from their production, while, at the same time point, the cecal luminal contents were obtained from the ceca immediately after mice were sacrificed. Building on all metagenomic and metaproteomic data reported here, light might be shed on the physical and biochemical variables that may account for the observed modification in structure and functions of the microbiota during the route from the cecum to the external environment. A first, key variable is the presence of oxygen. Clostridia cannot survive in oxic conditions, and their growth rate is critically reduced in microxic conditions (Al-Qadiri et al., 2015). In addition to spore formation, aerobiosis leads clostridia to develop L-forms, a condition characterized by the arrest of cell wall construction due to yet unknown changes in biochemical pathways (Mearls et al., 2012). On the other hand, oxygen stress triggers a complex and controlled response in *Bacteroides* spp., allowing their survival and persistence thanks to enzymatic reduction of oxygen-derived species via scavenging enzymes (including thioredoxin reductase, catalase, superoxide dismutase, peroxiredoxins, as observed in this study) (Rocha et al., 2007). Thus, given the metabolic features of these microbial groups, the growth rate of species belonging to anaerobic Firmicutes appears lower than that of Bacteroidetes species when the microbial community is exposed to the microxic milieu of the rectum and, then, to the oxic environment of the fecal sample. This may therefore be one of the reasons why Bacteroidetes exhibit a relative higher persistence in fecal samples compared to Firmicutes. The exposure to oxygen may also explain the different behavior of the facultative anaerobic or microaerophilic *Lactobacillus* spp., compared to the other Firmicutes genera, as well as the relatively higher abundance of certain genera belonging to facultative anaerobic Proteobacteria.

Another stress condition encountered by the microbiota when moving from the luminal cecum to the rectum is the reduction of water content, and the consequent variation in salt concentration. Both Gram-positive and -negative bacteria are known to orchestrate a response to osmotic shock that includes the accumulation of compatible solutes such as glutamate (Botsford et al., 1994). In keeping with this previous knowledge, we report here that Bacteroidetes functions related to glutamate synthesis (i.e., glutaminase and glutamate dehydrogenase) increase their relative abundance in the fecal samples compared to CCs. Another enzyme involved in glutamate metabolism, glutamate decarboxylase (GAD), is dramatically increased in the mice fecal samples. This variation is compatible with a pH drop in the fecal samples compared to the distal colon (Lewis and Heaton, 1997), since this enzyme allows the bacteria to maintain favorable intracellular pH conditions by optimizing availability of glutamate that, in turn, is converted to GABA by GAD, a reaction that increases the consumption of intracellular protons (Feehily and Karatzas, 2013).

Furthermore, our analysis of the KEGG Orthology functional groups showed an impressive and significant differential abundance of formyl-CoA transferase and oxalyl-CoA decarboxylase between CC and F metaproteome. These are the two key enzymes responsible for oxalotrophy, i.e., the ability to use oxalate as energy source as a result of bacterial catabolism. Oxalate is present in environments as diverse as soils or gastrointestinal tracts. Our data show an active role of Bacteroidetes in oxalate degradation in the fecal microbiome, in contrast with previous reports stating that oxalate-degrading bacteria are essentially restricted to three phyla, namely Actinobacteria, Firmicutes, and Proteobacteria (Herve et al., 2016). It needs to be noted, however, that most of the Bacteroidetes formyl-CoA transferase and oxalyl-CoA decarboxylase sequences matching with those identified in this study were added to the UniProt repository only in the last few years. Consistently with our results, 50 OTUs belonging to the uncultured Bacteroidales family S24-7 and detected in the woodrat gut microbiota were found to correlate significantly with oxalate consumption (Miller et al., 2016). The reason for this relatively increased abundance of oxalate degradation is not clear, since our experimental approach did not include specific metabolite analyses. A possible explanation might be found in the increased abundance in feces of Ascomycota (as clearly measured by S-MG and, specifically concerning Saccharomycetaceae, also by MP), as some members of this phylum are known to actively produce oxalate (Guimaraes and Stotz, 2004). In turn, Bacteroidetes survival and replication in the fecal sample would benefit by the degradation of this organic acid. A number of studies have pointed out that the extent of oxalate degradation in the intestine by the resident microbiota has an important impact on oxalate absorption and excretion (Li et al., 2015), as well as on crystal formation in the urinary tract (urolithiasis) (Knight et al., 2013). In addition to *Oxalobacter* and *Bifidobacterium* spp., Bacteroidetes might also be involved in these processes given their capability to degrade oxalate.

## Conclusion

This metaproteogenomic study allowed the identification of taxon-specific functions and metabolic pathways significantly differing in activity between the cecal luminal microbiota and the fecal microbiota. Our results open the way to new and deeper metaproteogenomic investigations aimed at elucidating functional dynamics of the microbial communities inhabiting the intestinal tract.

## Author Contributions

AT conceived the study, performed metaproteomics sample preparation and data analysis, supervised global data analysis and interpretation and wrote the manuscript. VM and CF performed metagenomic experiments and 16S metagenomics data analysis. AP performed metaproteomics sample preparation and mass spectrometry analysis. MA performed shotgun metagenomics data analysis and contributed to critically revise the manuscript. MD contributed to metagenomic experiments. MS performed mouse sample preparation and contributed to critically revise the manuscript. SU conceived the study, contributed to data interpretation and wrote the manuscript. All authors read and approved the final version of the manuscript.

## Conflict of Interest Statement

The authors declare that the research was conducted in the absence of any commercial or financial relationships that could be construed as a potential conflict of interest.

## Footnotes

1 ftp://ftp.ncbi.nlm.nih.gov/pub/UniVec

2 http://www.drive5.com/usearch/manual/otu_clustering.html

3 http://biit.cs.ut.ee/clustvis

4 https://inkscape.org

5 https://omics.pnl.gov/software/venn-diagram-plotter
