# Supplementary material for: Metaproteogenomics Reveals Taxonomic and Functional Changes between Cecal and Fecal Microbiota in Mouse
Source: Front Microbiol. 2017 Mar 14;8:391. doi: 10.3389/fmicb.2017.00391 (PMC5348496; doi:10.3389/fmicb.2017.00391)
Supplement: Supplementary file 1 [file Table_1.pdf]

Supplementary Tables

Metaproteogenomics reveals taxonomic and functional changes between cecal and fecal microbiota in mouse

Alessandro Tanca, Valeria Manghina, Cristina Fraumene, Antonio Palomba, Marcello Abbondio, Massimo Deligios, Michael Silverman, and Sergio Uzzau*

*** Correspondence:** Sergio Uzzau: uzzau@portocontericerche.it

Table S1. Functional-taxonomic features with significantly differential abundance between cecal and fecal metagenome.

KEGG number	function	phylum	adjusted *p*-value	mean fold-change (CC/F)	SEM
K01198	xylan 1,4-beta-xylosidase	Firmicutes	6.9E-249	13.06	12.50
K00767	nicotinate-nucleotide pyrophosphorylase (carboxylating)	Firmicutes	1.4E-268	12.66	10.92
K00557	tRNA (uracil-5-)-methyltransferase	Firmicutes	3.8E-264	11.63	9.69
K15898	pseudaminic acid synthase	Firmicutes	1.1E-269	10.96	9.28
K05352	ribitol-5-phosphate 2-dehydrogenase	Firmicutes	7.8E-308	9.73	5.78
K15532	unsaturated rhamnogalacturonyl hydrolase	Firmicutes	7.8E-308	8.77	6.12
K03517	quinolinate synthase	Firmicutes	2.2E-218	8.06	5.63
K00048	lactaldehyde reductase	Firmicutes	3.8E-37	6.90	5.13
K10119	raffinose/stachyose/melibiose transport system permease protein	Firmicutes	4.4E-225	6.63	4.69
K00794	6,7-dimethyl-8-ribityllumazine synthase	Firmicutes	2.2E-285	6.57	3.97
K15739	D-alanine---(R)-lactate ligase	Firmicutes	1.2E-144	6.55	4.73
K18205	non-reducing end beta-L-arabinofuranosidase	Firmicutes	4.0E-252	6.45	4.04
K01666	4-hydroxy 2-oxovalerate aldolase	Firmicutes	7.8E-308	6.33	1.64
K13010	perosamine synthetase	Firmicutes	1.0E-57	5.85	4.33
K01625	2-dehydro-3-deoxyphosphogluconate aldolase / (4S)-4-hydroxy-2-oxoglutarate aldolase	Firmicutes	1.0E-34	5.79	4.00
K00684	leucyl/phenylalanyl-tRNA--protein transferase	Firmicutes	4.9E-153	5.70	5.23
K00690	sucrose phosphorylase	Firmicutes	7.8E-308	5.64	1.46
K00540	[no name]	Firmicutes	1.5E-71	5.56	3.42
K03410	chemotaxis protein CheC	Firmicutes	2.4E-231	5.50	3.50
K03521	electron transfer flavoprotein beta subunit	Firmicutes	6.9E-143	5.22	2.89
K01611	S-adenosylmethionine decarboxylase	Firmicutes	7.8E-308	5.08	1.23
K12372	dipeptide transport system ATP-binding protein	Firmicutes	7.8E-308	5.07	1.43
K16907	fluoroquinolone transport system ATP-binding protein	Firmicutes	3.0E-160	4.91	2.47
K03500	16S rRNA (cytosine967-C5)-methyltransferase	Firmicutes	5.3E-81	4.89	3.06
K07576	metallo-beta-lactamase family protein	Firmicutes	3.9E-138	4.84	2.55
K03408	purine-binding chemotaxis protein CheW	Firmicutes	7.8E-308	4.81	1.82
K03320	ammonium transporter, Amt family	Firmicutes	1.6E-243	4.74	2.10

KEGG number	function	phylum	adjusted *p*-value	mean fold-change (CC/F)	SEM
K02338	DNA polymerase III subunit beta	Firmicutes	7.6E-261	4.72	1.78
K02488	two-component system, cell cycle response regulator	Firmicutes	1.6E-162	4.55	2.51
K03271	D-sedoheptulose 7-phosphate isomerase	Firmicutes	7.5E-94	4.52	2.54
K00335	NADH-quinone oxidoreductase subunit F	Firmicutes	5.1E-67	4.48	2.51
K00209	enoyl-[acyl-carrier protein] reductase / trans-2-enoyl-CoA reductase (NAD+)	Firmicutes	7.8E-308	4.44	0.87
K00140	malonate-semialdehyde dehydrogenase (acetylating) / methylmalonate-semialdehyde dehydrogenase	Firmicutes	7.8E-308	4.33	1.59
K06148	ATP-binding cassette, subfamily C, bacterial	Firmicutes	7.8E-308	4.23	0.92
K07402	xanthine dehydrogenase accessory factor	Firmicutes	8.4E-293	4.17	1.76
K01598	phosphopantothenoylcysteine decarboxylase	Firmicutes	7.8E-308	4.17	0.93
K01811	alpha-D-xyloside xylohydrolase	Firmicutes	5.9E-177	4.14	1.40
K03656	ATP-dependent DNA helicase Rep	Firmicutes	4.9E-186	4.12	1.70
K01711	GDPmannose 4,6-dehydratase	Proteobacteria	1.8E-93	4.11	2.69
K07301	cation:H+ antiporter	Firmicutes	2.7E-62	4.04	2.23
K18122	4-hydroxybutyrate CoA-transferase	Firmicutes	3.3E-122	4.00	1.25
K01007	pyruvate, water dikinase	Firmicutes	7.9E-285	3.98	1.19
K02058	simple sugar transport system substrate-binding protein	Firmicutes	3.3E-259	3.98	1.49
K07742	uncharacterized protein	Firmicutes	7.8E-308	3.97	1.38
K11634	two-component system, OmpR family, response regulator YxdJ	Firmicutes	2.3E-104	3.97	1.80
K03426	NAD+ diphosphatase	Firmicutes	2.5E-193	3.83	1.83
K07696	two-component system, NarL family, response regulator NreC	Firmicutes	7.8E-308	3.83	1.20
K00167	2-oxoisovalerate dehydrogenase E1 component beta subunit	Firmicutes	1.8E-187	3.82	1.59
K11752	diaminohydroxyphosphoribosylamino pyrimidine deaminase / 5-amino-6-(5-phosphoribosylamino)uracil reductase	Firmicutes	2.8E-300	3.78	1.25
K16951	anaerobic sulfite reductase subunit B	Firmicutes	7.8E-308	3.78	0.64
K02224	cobyrinic acid a,c-diamide synthase	Firmicutes	4.2E-184	3.77	1.25
K03778	D-lactate dehydrogenase	Firmicutes	7.8E-308	3.76	0.92
K06284	transcriptional pleiotropic regulator of transition state genes	Firmicutes	4.8E-251	3.75	1.19
K00262	glutamate dehydrogenase (NADP+)	Firmicutes	3.8E-183	3.74	1.34

KEGG number	function	phylum	adjusted *p*-value	mean fold-change (CC/F)	SEM
K06871	uncharacterized protein	Firmicutes	7.8E-308	3.71	0.65
K12297	23S rRNA (guanine2445-N2)-methyltransferase / 23S rRNA (guanine2069-N7)-methyltransferase	Firmicutes	6.9E-72	3.71	1.66
K03476	L-ascorbate 6-phosphate lactonase	Firmicutes	1.5E-73	3.66	1.10
K07775	two-component system, OmpR family, response regulator ResD	Firmicutes	1.4E-244	3.62	0.98
K10440	ribose transport system permease protein	Firmicutes	7.8E-308	3.60	0.64
K01657	anthranilate synthase component I	Firmicutes	5.1E-257	3.58	1.09
K06407	stage V sporulation protein AE	Firmicutes	5.1E-142	3.58	1.37
K06217	phosphate starvation-inducible protein PhoH and related proteins	Firmicutes	1.0E-15	3.57	1.53
K06408	stage V sporulation protein AF	Firmicutes	5.2E-53	3.52	1.08
K10548	putative multiple sugar transport system ATP-binding protein	Firmicutes	3.3E-166	3.52	1.17
K18785	beta-1,4-mannooligosaccharide/beta-1,4-mannosyl-N-acetylglucosamine phosphorylase	Firmicutes	8.3E-151	3.52	1.11
K00956	sulfate adenylyltransferase subunit 1	Firmicutes	2.0E-50	3.50	1.32
K09809	CDP-glycerol glycerophosphotransferase	Firmicutes	7.8E-308	3.48	0.70
K00957	sulfate adenylyltransferase subunit 2	Firmicutes	1.9E-163	3.42	0.90
K03621	glycerol-3-phosphate acyltransferase PlsX	Firmicutes	7.8E-193	3.42	1.07
K02420	flagellar biosynthetic protein FliQ	Firmicutes	6.0E-294	3.42	0.73
K01209	alpha-N-arabinofuranosidase	Firmicutes	7.8E-308	3.41	0.31
K01749	hydroxymethylbilane synthase	Firmicutes	4.6E-17	3.37	2.46
K00634	phosphate butyryltransferase	Firmicutes	7.8E-308	3.36	0.45
K18345	two-component system, OmpR family, sensor histidine kinase VanS	Firmicutes	7.3E-301	3.33	0.60
K04562	flagellar biosynthesis protein FlhG	Firmicutes	7.8E-308	3.33	0.93
K17320	putative aldouronate transport system permease protein	Firmicutes	3.5E-279	3.31	0.68
K03080	L-ribulose-5-phosphate 4-epimerase	Firmicutes	1.2E-258	3.30	0.86
K02879	large subunit ribosomal protein L17	Firmicutes	8.2E-194	3.28	0.85
K02474	UDP-N-acetyl-D-galactosamine dehydrogenase	Firmicutes	4.4E-306	3.27	0.54
K02025	multiple sugar transport system permease protein	Firmicutes	2.2E-255	3.27	0.81
K00872	homoserine kinase	Firmicutes	3.0E-296	3.25	0.63
K06972	presequence protease	Firmicutes	1.2E-173	3.23	0.77
K00031	isocitrate dehydrogenase	Firmicutes	7.8E-308	3.23	0.60

KEGG number	function	phylum	adjusted *p*-value	mean fold-change (CC/F)	SEM
K07664	two-component system, OmpR family, response regulator BaeR	Firmicutes	1.3E-236	3.23	0.67
K01647	citrate synthase	Firmicutes	1.0E-140	3.20	1.06
K01182	oligo-1,6-glucosidase	Firmicutes	3.2E-05	3.20	1.30
K10017	histidine transport system ATP-binding protein	Firmicutes	7.8E-308	3.18	0.32
K00090	gluconate 2-dehydrogenase	Firmicutes	7.2E-232	3.17	0.73
K09684	purine catabolism regulatory protein	Firmicutes	8.0E-297	3.17	0.73
K00991	2-C-methyl-D-erythritol 4-phosphate cytidylyltransferase	Firmicutes	7.2E-10	3.16	1.26
K16792	methanogen homoaconitase large subunit	Firmicutes	2.4E-67	3.16	1.35
K07114	Ca-activated chloride channel homolog	Firmicutes	1.3E-302	3.16	0.34
K01496	phosphoribosyl-AMP cyclohydrolase	Firmicutes	4.6E-61	3.14	0.87
K01153	type I restriction enzyme, R subunit	Firmicutes	3.2E-02	3.14	0.97
K05878	dihydroxyacetone kinase, N-terminal domain	Firmicutes	2.9E-239	3.13	0.57
K00796	dihydropteroate synthase	Firmicutes	9.4E-08	3.12	1.03
K05877	methyl-accepting chemotaxis protein IV, peptide sensor receptor	Firmicutes	5.5E-128	3.10	0.78
K18220	ribosomal protection tetracycline resistance protein	Firmicutes	6.6E-126	3.09	0.98
K05349	beta-glucosidase	Firmicutes	2.2E-121	3.07	0.95
K03546	exonuclease SbcC	Firmicutes	1.7E-277	3.04	0.63
K01693	imidazoleglycerol-phosphate dehydratase	Firmicutes	1.3E-161	3.04	0.89
K02356	elongation factor P	Firmicutes	3.6E-156	3.03	0.71
K03783	purine-nucleoside phosphorylase	Firmicutes	7.8E-308	3.03	0.44
K13653	AraC family transcriptional regulator	Firmicutes	8.6E-195	3.01	0.68
K09814	hemin transport system ATP-binding protein	Firmicutes	5.0E-123	3.00	0.87
K13497	anthranilate synthase/phosphoribosyltransferase	Firmicutes	5.5E-121	3.00	0.87
K11923	MerR family transcriptional regulator, copper efflux regulator	Firmicutes	7.8E-308	3.00	0.87
K00052	3-isopropylmalate dehydrogenase	Proteobacteria	7.8E-308	3.00	0.76
K19575	MerR family transcriptional regulator, activator of bmr gene	Firmicutes	2.7E-260	3.00	0.73
K03522	electron transfer flavoprotein alpha subunit	Firmicutes	7.8E-308	2.99	0.39
K01235	alpha-glucuronidase	Firmicutes	6.4E-233	2.97	0.92

KEGG number	function	phylum	adjusted *p*-value	mean fold-change (CC/F)	SEM
K01825	3-hydroxyacyl-CoA dehydrogenase / enoyl-CoA hydratase / 3-hydroxybutyryl-CoA epimerase / enoyl-CoA isomerase	Firmicutes	2.1E-07	2.94	0.92
K06200	carbon starvation protein	Firmicutes	3.4E-182	2.94	0.79
K01495	GTP cyclohydrolase I	Firmicutes	2.5E-26	2.94	0.95
K03327	multidrug resistance protein, MATE family	Firmicutes	2.0E-189	2.93	0.64
K15583	oligopeptide transport system ATP-binding protein	Firmicutes	7.8E-308	2.92	0.86
K11070	spermidine/putrescine transport system permease protein	Firmicutes	3.1E-210	2.92	0.79
K02902	large subunit ribosomal protein L28	Firmicutes	7.8E-308	2.90	0.37
K03465	thymidylate synthase (FAD)	Firmicutes	2.4E-248	2.90	0.38
K01960	pyruvate carboxylase subunit B	Firmicutes	7.8E-308	2.88	0.49
K02502	ATP phosphoribosyltransferase regulatory subunit	Firmicutes	1.0E-45	2.86	0.80
K00929	butyrate kinase	Firmicutes	1.3E-225	2.85	0.54
K01926	redox-sensing transcriptional repressor	Firmicutes	1.9E-13	2.84	0.85
K07095	uncharacterized protein	Firmicutes	7.8E-308	2.83	0.88
K10440	ribose transport system permease protein	Proteobacteria	1.0E-238	2.83	1.92
K02669	twitching motility protein PilT	Firmicutes	3.3E-228	2.83	0.50
K01786	L-ribulose-5-phosphate 4-epimerase	Firmicutes	1.2E-258	2.81	0.60
K00640	serine O-acetyltransferase	Firmicutes	5.9E-119	2.81	0.67
K11928	sodium/proline symporter	Firmicutes	1.1E-252	2.80	0.47
K02416	flagellar motor switch protein FliM	Firmicutes	8.0E-216	2.80	0.72
K16201	dipeptide transport system permease protein	Firmicutes	1.5E-15	2.80	0.89
K05593	aminoglycoside 6-adenylyltransferase	Firmicutes	5.7E-87	2.79	0.48
K02802	PTS system, N-acetylglucosamine-specific IIA component	Firmicutes	1.5E-76	2.79	0.87
K00831	phosphoserine aminotransferase	Firmicutes	7.8E-308	2.79	0.19
K00053	ketol-acid reductoisomerase	Firmicutes	7.8E-308	2.78	0.32
K00975	glucose-1-phosphate adenylyltransferase	Firmicutes	3.5E-97	2.77	0.79
K13626	flagellar assembly factor FliW	Firmicutes	6.2E-277	2.77	0.22
K09693	teichoic acid transport system ATP-binding protein	Firmicutes	4.8E-15	2.76	0.85
K03074	preprotein translocase subunit SecF	Firmicutes	2.8E-155	2.75	0.72
K03339	6-phospho-5-dehydro-2-deoxy-D-gluconate aldolase	Firmicutes	2.6E-43	2.74	0.88

KEGG number	function	phylum	adjusted *p*-value	mean fold-change (CC/F)	SEM
K01815	4-deoxy-L-threo-5-hexosulose-uronate ketol-isomerase	Firmicutes	7.8E-308	2.74	0.23
K02763	PTS system, D-glucosamine-specific IIA component	Firmicutes	5.2E-176	2.74	0.63
K07571	S1 RNA binding domain protein	Firmicutes	2.4E-32	2.73	0.87
K00974	tRNA nucleotidyltransferase (CCA-adding enzyme)	Firmicutes	1.5E-12	2.72	0.95
K00248	butyryl-CoA dehydrogenase	Firmicutes	7.8E-308	2.71	0.41
K16213	cellobiose epimerase	Firmicutes	1.2E-91	2.70	0.68
K06023	HPr kinase/phosphorylase	Firmicutes	3.3E-15	2.69	0.86
K00860	adenylylsulfate kinase	Firmicutes	6.0E-221	2.69	0.17
K07771	two-component system, OmpR family, response regulator BasR	Firmicutes	7.4E-140	2.68	0.69
K12984	(heptosyl)LPS beta-1,4-glucosyltransferase	Firmicutes	3.5E-132	2.68	0.63
K02124	V/A-type H+/Na+-transporting ATPase subunit K	Firmicutes	6.2E-189	2.67	2.03
K01069	hydroxyacylglutathione hydrolase	Firmicutes	7.8E-308	2.67	0.44
K13731	internalin B	Firmicutes	7.8E-308	2.67	0.93
K02406	flagellin	Firmicutes	1.5E-74	2.67	0.66
K02529	LacI family transcriptional regulator	Firmicutes	3.1E-23	2.66	0.78
K10234	alpha-glucoside transport system permease protein	Firmicutes	1.3E-103	2.66	0.54
K02913	large subunit ribosomal protein L33	Firmicutes	2.4E-271	2.65	0.44
K10542	methyl-galactoside transport system ATP-binding protein	Firmicutes	1.3E-13	2.65	0.90
K02967	small subunit ribosomal protein S2	Firmicutes	5.5E-169	2.65	0.56
K07679	two-component system, NarL family, sensor histidine kinase EvgS	Firmicutes	1.9E-142	2.65	0.75
K13815	two-component system, response regulator RpfG	Proteobacteria	1.2E-18	2.64	0.93
K02400	flagellar biosynthesis protein FlhA	Firmicutes	7.8E-308	2.63	0.04
K10562	rhamnose transport system ATP-binding protein	Firmicutes	3.3E-16	2.63	0.94
K10546	putative multiple sugar transport system substrate-binding protein	Firmicutes	6.1E-262	2.61	0.26
K17319	putative aldouronate transport system permease protein	Firmicutes	3.5E-279	2.61	0.55
K07714	two-component system, NtrC family, response regulator AtoC	Firmicutes	1.8E-08	2.61	0.96
K00666	fatty-acyl-CoA synthase	Firmicutes	5.8E-286	2.61	0.07
K02115	F-type H+-transporting ATPase subunit gamma	Firmicutes	3.0E-307	2.60	0.41
K03595	GTPase	Firmicutes	2.0E-249	2.59	0.61

KEGG number	function	phylum	adjusted *p*-value	mean fold-change (CC/F)	SEM
K10111	multiple sugar transport system ATP-binding protein	Firmicutes	7.8E-308	2.58	0.58
K02867	large subunit ribosomal protein L11	Firmicutes	1.2E-226	2.57	0.47
K01624	fructose-bisphosphate aldolase, class II	Firmicutes	2.0E-23	2.56	0.77
K02392	flagellar basal-body rod protein FlgG	Firmicutes	5.8E-250	2.56	0.47
K01523	phosphoribosyl-ATP pyrophosphohydrolase	Firmicutes	2.9E-196	2.56	0.50
K00973	glucose-1-phosphate thymidylyltransferase	Firmicutes	1.3E-122	2.56	0.54
K11071	spermidine/putrescine transport system permease protein	Firmicutes	3.1E-210	2.55	0.64
K00788	thiamine-phosphate pyrophosphorylase	Firmicutes	6.0E-69	2.55	0.66
K01803	triosephosphate isomerase (TIM)	Firmicutes	7.8E-28	2.54	0.73
K06001	tryptophan synthase beta chain	Firmicutes	4.0E-299	2.54	0.34
K03654	ATP-dependent DNA helicase RecQ	Firmicutes	6.2E-101	2.54	0.60
K01966	propionyl-CoA carboxylase beta chain	Firmicutes	9.3E-197	2.54	0.63
K12410	NAD-dependent deacetylase	Firmicutes	7.8E-308	2.54	0.29
K12371	dipeptide transport system ATP-binding protein	Firmicutes	7.8E-308	2.53	0.30
K01886	glutaminyl-tRNA synthetase	Firmicutes	7.8E-308	2.53	0.28
K00954	pantetheine-phosphate adenylyltransferase	Firmicutes	1.4E-141	2.52	0.52
K00087	xanthine dehydrogenase molybdenum-binding subunit	Firmicutes	1.2E-44	2.52	0.74
K10907	aminotransferase	Firmicutes	7.8E-308	2.52	0.30
K02988	small subunit ribosomal protein S5	Firmicutes	2.3E-164	2.52	0.53
K01940	argininosuccinate synthase	Firmicutes	7.8E-308	2.52	0.33
K00074	3-hydroxybutyryl-CoA dehydrogenase	Firmicutes	7.8E-308	2.52	0.22
K01866	tyrosyl-tRNA synthetase	Firmicutes	2.0E-40	2.51	0.61
K00791	tRNA dimethylallyltransferase	Firmicutes	1.2E-07	2.51	0.71
K01809	mannose-6-phosphate isomerase	Firmicutes	1.1E-295	2.51	0.37
K00013	histidinol dehydrogenase	Firmicutes	5.2E-268	2.50	0.44
K11069	spermidine/putrescine transport system substrate-binding protein	Firmicutes	2.5E-115	2.50	0.43
K02500	cyclase	Proteobacteria	1.1E-203	2.50	0.29
K01154	type I restriction enzyme, S subunit	Firmicutes	7.8E-308	2.50	0.58
K00111	glycerol-3-phosphate dehydrogenase	Firmicutes	4.7E-278	2.50	1.00
K00937	polyphosphate kinase	Firmicutes	1.7E-234	2.50	0.48
K01867	tryptophanyl-tRNA synthetase	Firmicutes	4.2E-06	2.49	0.71
K00882	1-phosphofructokinase	Firmicutes	3.3E-90	2.49	0.59

KEGG number	function	phylum	adjusted *p*-value	mean fold-change (CC/F)	SEM
K00765	ATP phosphoribosyltransferase	Firmicutes	8.8E-214	2.48	0.49
K01734	methylglyoxal synthase	Firmicutes	7.8E-308	2.48	0.36
K00850	6-phosphofructokinase 1	Firmicutes	5.5E-46	2.48	0.66
K00702	cellobiose phosphorylase	Firmicutes	6.0E-20	2.47	0.75
K07663	two-component system, OmpR family, catabolic regulation response regulator CreB	Firmicutes	3.4E-85	2.47	0.72
K03230	type III secretion protein V	Firmicutes	2.7E-91	2.47	0.58
K01923	phosphoribosylaminoimidazole-succinocarboxamide synthase	Firmicutes	1.5E-235	2.46	0.41
K01812	glucuronate isomerase	Firmicutes	7.8E-308	2.44	0.19
K00762	orotate phosphoribosyltransferase	Firmicutes	1.2E-06	2.43	0.70
K09121	uncharacterized protein	Firmicutes	7.8E-308	2.43	0.68
K03412	two-component system, chemotaxis family, response regulator CheB	Firmicutes	4.5E-162	2.43	0.55
K01515	ADP-ribose pyrophosphatase	Firmicutes	1.0E-137	2.42	0.08
K01424	L-asparaginase	Firmicutes	1.0E-52	2.42	0.61
K01649	2-isopropylmalate synthase	Firmicutes	7.8E-308	2.42	0.20
K02876	large subunit ribosomal protein L15	Firmicutes	6.6E-71	2.41	0.52
K15524	mannosylglycerate hydrolase	Firmicutes	6.4E-150	2.41	0.53
K01615	glutaconyl-CoA decarboxylase	Firmicutes	7.8E-308	2.41	0.11
K01711	GDPmannose 4,6-dehydratase	Firmicutes	2.4E-192	2.41	0.51
K03978	GTP-binding protein	Firmicutes	1.5E-185	2.41	0.43
K02887	large subunit ribosomal protein L20	Firmicutes	9.4E-212	2.40	0.42
K04763	integrase/recombinase XerD	Firmicutes	1.3E-79	2.40	0.51
K07260	D-alanyl-D-alanine carboxypeptidase	Firmicutes	7.2E-08	2.40	0.66
K02390	flagellar hook protein FlgE	Firmicutes	7.8E-308	2.40	0.28
K00848	rhamnulokinase	Firmicutes	1.1E-283	2.40	0.30
K01760	cystathionine beta-lyase	Firmicutes	6.0E-41	2.40	0.71
K03671	thioredoxin 1	Firmicutes	7.4E-192	2.40	0.47
K00611	ornithine carbamoyltransferase	Firmicutes	7.8E-308	2.40	0.30
K16212	4-O-beta-D-mannosyl-D-glucose phosphorylase	Firmicutes	4.4E-125	2.39	0.45
K00648	3-oxoacyl-[acyl-carrier-protein] synthase III	Firmicutes	2.6E-03	2.39	0.61
K07768	two-component system, OmpR family, sensor histidine kinase SenX3	Firmicutes	1.4E-53	2.39	0.49
K03816	xanthine phosphoribosyltransferase	Firmicutes	7.8E-04	2.39	0.63
K07648	two-component system, OmpR family, aerobic respiration control sensor histidine kinase ArcB	Firmicutes	1.0E-134	2.39	0.45
K12146	hydrogenase-4 transcriptional activator	Firmicutes	3.1E-129	2.39	0.39
K02945	small subunit ribosomal protein S1	Firmicutes	1.4E-51	2.39	0.54

KEGG number	function	phylum	adjusted *p*-value	mean fold-change (CC/F)	SEM
K15915	undecaprenyl phosphate N,N'-diacetylbacillosamine 1-phosphate transferase	Firmicutes	7.6E-217	2.38	0.49
K02057	simple sugar transport system permease protein	Firmicutes	4.0E-174	2.38	0.55
K00826	branched-chain amino acid aminotransferase	Firmicutes	7.8E-308	2.38	0.30
K01572	oxaloacetate decarboxylase, beta subunit	Firmicutes	7.8E-308	2.37	0.32
K00939	adenylate kinase	Firmicutes	6.0E-35	2.37	0.54
K02410	flagellar motor switch protein FliG	Firmicutes	2.1E-219	2.37	0.46
K10773	endonuclease III	Firmicutes	1.7E-222	2.37	0.45
K00955	bifunctional enzyme CysN/CysC	Firmicutes	6.1E-117	2.37	0.19
K06941	23S rRNA (adenine2503-C2)-methyltransferase	Firmicutes	2.4E-159	2.36	0.45
K07658	two-component system, OmpR family, alkaline phosphatase synthesis response regulator PhoP	Firmicutes	5.6E-222	2.36	0.45
K02120	V/A-type H+/Na+-transporting ATPase subunit D	Firmicutes	3.3E-204	2.35	0.47
K03976	Cys-tRNA(Pro)/Cys-tRNA(Cys) deacylase	Firmicutes	2.0E-281	2.35	0.28
K01715	enoyl-CoA hydratase	Firmicutes	7.8E-308	2.35	0.16
K00705	4-alpha-glucanotransferase	Firmicutes	4.1E-26	2.35	0.53
K16786	energy-coupling factor transport system ATP-binding protein	Firmicutes	6.8E-77	2.34	0.48
K08722	5'-deoxynucleotidase	Firmicutes	4.0E-226	2.34	0.33
K07667	two-component system, OmpR family, KDP operon response regulator KdpE	Firmicutes	2.6E-13	2.34	0.58
K04759	ferrous iron transport protein B	Firmicutes	7.8E-308	2.34	0.28
K07139	uncharacterized protein	Firmicutes	7.8E-308	2.34	0.61
K13918	glucarate dehydratase-related protein	Firmicutes	3.1E-108	2.33	0.38
K03785	3-dehydroquinate dehydratase I	Proteobacteria	3.1E-288	2.33	0.44
K01955	carbamoyl-phosphate synthase large subunit	Spirochaetes	2.1E-297	2.33	0.17
K00760	hypoxanthine phosphoribosyltransferase	Firmicutes	8.4E-06	2.33	0.57
K01784	UDP-glucose 4-epimerase	Firmicutes	1.1E-205	2.33	0.41
K00052	3-isopropylmalate dehydrogenase	Firmicutes	7.8E-308	2.33	0.28
K06182	23S rRNA pseudouridine2604 synthase	Firmicutes	4.5E-185	2.33	0.44
K00088	IMP dehydrogenase	Firmicutes	1.9E-242	2.32	0.39

KEGG number	function	phylum	adjusted *p*-value	mean fold-change (CC/F)	SEM
K18349	two-component system, OmpR family, response regulator VanR	Firmicutes	4.5E-145	2.32	0.46
K02483	two-component system, OmpR family, response regulator	Firmicutes	6.7E-39	2.32	0.53
K02417	flagellar motor switch protein FliN/FliY	Firmicutes	5.1E-302	2.32	0.34
K03625	N utilization substance protein B	Firmicutes	9.0E-230	2.32	0.44
K02372	3-hydroxyacyl-[acyl-carrier-protein] dehydratase	Firmicutes	5.9E-31	2.31	0.63
K10545	D-xylose transport system ATP-binding protein	Firmicutes	5.6E-158	2.31	0.47
K02837	peptide chain release factor 3	Firmicutes	2.2E-293	2.30	0.30
K15022	formate dehydrogenase beta subunit	Firmicutes	9.7E-211	2.30	0.43
K06595	heam-based aerotactic trancducer	Firmicutes	3.5E-110	2.30	0.15
K01839	phosphopentomutase	Firmicutes	1.3E-183	2.30	0.38
K00688	starch phosphorylase	Firmicutes	3.3E-203	2.30	0.38
K02909	large subunit ribosomal protein L31	Firmicutes	3.5E-303	2.29	0.28
K02926	large subunit ribosomal protein L4	Firmicutes	7.8E-308	2.29	0.28
K02970	small subunit ribosomal protein S21	Firmicutes	1.6E-168	2.28	0.44
K14982	two-component system, OmpR family, sensor histidine kinase CiaH	Firmicutes	5.1E-133	2.28	0.33
K01665	para-aminobenzoate synthetase component I	Firmicutes	1.4E-148	2.28	0.15
K02112	F-type H+-transporting ATPase subunit beta	Firmicutes	2.3E-111	2.27	0.45
K07042	probable rRNA maturation factor	Firmicutes	4.7E-244	2.27	0.38
K00548	5-methyltetrahydrofolate--homocysteine methyltransferase	Firmicutes	9.4E-105	2.27	0.51
K02036	phosphate transport system ATP-binding protein	Firmicutes	6.1E-18	2.27	0.54
K03101	signal peptidase II	Firmicutes	3.1E-17	2.26	0.62
K01356	repressor LexA	Firmicutes	4.8E-224	2.25	0.35
K14441	ribosomal protein S12 methylthiotransferase	Firmicutes	2.0E-220	2.25	0.35
K01686	mannonate dehydratase	Firmicutes	7.8E-308	2.25	0.19
K01755	argininosuccinate lyase	Firmicutes	6.6E-164	2.25	0.38
K19425	glycosyltransferase EpsH	Firmicutes	1.1E-66	2.25	0.53
K03564	peroxiredoxin Q/BCP	Firmicutes	1.3E-03	2.24	0.61
K02904	large subunit ribosomal protein L29	Firmicutes	7.8E-308	2.24	0.18
K00620	glutamate N-acetyltransferase / amino-acid N-acetyltransferase	Firmicutes	7.8E-308	2.24	0.24
K02111	F-type H+-transporting ATPase subunit alpha	Firmicutes	6.3E-154	2.23	0.40

KEGG number	function	phylum	adjusted *p*-value	mean fold-change (CC/F)	SEM
K02013	iron complex transport system ATP-binding protein	Firmicutes	1.0E-207	2.23	0.39
K11074	putrescine transport system permease protein	Firmicutes	2.5E-95	2.23	0.45
K01951	GMP synthase (glutamine-hydrolysing)	Firmicutes	8.9E-43	2.23	0.46
K01159	crossover junction endodeoxyribonuclease RuvC	Firmicutes	9.5E-166	2.23	0.40
K10112	multiple sugar transport system ATP-binding protein	Firmicutes	7.8E-308	2.23	0.12
K01619	deoxyribose-phosphate aldolase	Firmicutes	2.9E-147	2.22	0.43
K02056	simple sugar transport system ATP-binding protein	Firmicutes	6.5E-131	2.22	0.42
K00354	NADPH2 dehydrogenase	Firmicutes	3.3E-75	2.22	0.28
K00703	starch synthase	Firmicutes	2.2E-29	2.22	0.49
K01872	alanyl-tRNA synthetase	Firmicutes	1.0E-208	2.22	0.35
K16787	energy-coupling factor transport system ATP-binding protein	Firmicutes	6.8E-77	2.21	0.45
K01246	DNA-3-methyladenine glycosylase I	Firmicutes	1.6E-157	2.21	0.12
K05816	sn-glycerol 3-phosphate transport system ATP-binding protein	Firmicutes	9.6E-198	2.20	0.41
K01938	formate--tetrahydrofolate ligase	Firmicutes	1.1E-12	2.20	0.54
K08998	uncharacterized protein	Firmicutes	7.8E-308	2.20	0.42
K02992	small subunit ribosomal protein S7	Firmicutes	1.1E-25	2.20	0.47
K02438	glycogen operon protein	Firmicutes	5.9E-07	2.20	0.49
K00980	glycerol-3-phosphate cytidylyltransferase	Firmicutes	1.5E-85	2.20	0.45
K00626	acetyl-CoA C-acetyltransferase	Firmicutes	1.2E-291	2.19	0.30
K04769	AbrB family transcriptional regulator, stage V sporulation protein T	Firmicutes	7.7E-90	2.19	0.41
K03613	electron transport complex protein RnfE	Firmicutes	7.8E-308	2.19	0.26
K02412	flagellum-specific ATP synthase	Firmicutes	7.8E-308	2.18	0.26
K03704	cold shock protein (beta-ribbon, CspA family)	Firmicutes	7.6E-85	2.18	0.42
K02963	small subunit ribosomal protein S18	Firmicutes	7.8E-308	2.18	0.25
K00046	gluconate 5-dehydrogenase	Firmicutes	1.5E-10	2.18	0.57
K03411	chemotaxis protein CheD	Firmicutes	2.9E-247	2.18	0.40
K02388	flagellar basal-body rod protein FlgC	Firmicutes	9.9E-247	2.18	0.15
K01491	methylenetetrahydrofolate dehydrogenase (NADP+) / methenyltetrahydrofolate cyclohydrolase	Firmicutes	8.5E-160	2.18	0.36

KEGG number	function	phylum	adjusted *p*-value	mean fold-change (CC/F)	SEM
K09710	ribosome-associated protein	Firmicutes	1.1E-166	2.18	0.23
K05910	NADH peroxidase	Firmicutes	1.4E-251	2.17	0.33
K03407	two-component system, chemotaxis family, sensor kinase CheA	Firmicutes	1.5E-282	2.17	0.33
K03413	two-component system, chemotaxis family, response regulator CheY	Firmicutes	5.1E-19	2.17	0.57
K02968	small subunit ribosomal protein S20	Firmicutes	2.5E-58	2.16	0.50
K01609	indole-3-glycerol phosphate synthase	Firmicutes	1.4E-263	2.15	0.34
K00891	shikimate kinase	Firmicutes	9.5E-260	2.15	0.17
K00700	1,4-alpha-glucan branching enzyme	Firmicutes	1.4E-273	2.15	0.29
K00600	glycine hydroxymethyltransferase	Firmicutes	1.4E-17	2.15	0.53
K02401	flagellar biosynthetic protein FlhB	Firmicutes	6.0E-290	2.15	0.30
K01687	dihydroxy-acid dehydratase	Firmicutes	7.8E-308	2.15	0.23
K02343	DNA polymerase III subunit gamma/tau	Firmicutes	3.9E-167	2.14	0.39
K05936	precorrin-4/cobalt-precorrin-4 C11-methyltransferase	Firmicutes	1.4E-04	2.14	0.54
K11085	ATP-binding cassette, subfamily B, bacterial MsbA	Firmicutes	8.7E-172	2.14	0.38
K02520	translation initiation factor IF-3	Firmicutes	7.1E-16	2.13	0.47
K00266	glutamate synthase (NADPH/NADH) small chain	Firmicutes	2.0E-123	2.12	0.42
K02888	large subunit ribosomal protein L21	Firmicutes	1.3E-68	2.12	0.44
K01704	3-isopropylmalate/(R)-2-methylmalate dehydratase small subunit	Firmicutes	3.8E-60	2.12	0.47
K10041	putative glutamine transport system ATP-binding protein	Firmicutes	5.0E-58	2.12	0.47
K03977	GTPase	Firmicutes	2.0E-249	2.12	0.47
K00950	2-amino-4-hydroxy-6-hydroxymethyldihydropteridine diphosphokinase	Firmicutes	1.1E-04	2.11	0.51
K05810	laccase domain-containing protein	Firmicutes	1.7E-28	2.11	0.51
K00876	uridine kinase	Firmicutes	2.1E-36	2.10	0.47
K01338	ATP-dependent Lon protease	Firmicutes	7.8E-308	2.10	0.21
K06206	sugar fermentation stimulation protein A	Firmicutes	6.3E-70	2.10	0.47
K01520	dUTP pyrophosphatase	Firmicutes	7.8E-308	2.10	0.13
K01090	protein phosphatase	Firmicutes	3.7E-04	2.09	0.59
K01129	dGTPase	Firmicutes	3.7E-39	2.09	0.45
K03526	(E)-4-hydroxy-3-methylbut-2-enyl-diphosphate synthase	Firmicutes	9.7E-07	2.09	0.46
K03569	rod shape-determining protein MreB and related proteins	Firmicutes	7.7E-60	2.09	0.39

KEGG number	function	phylum	adjusted *p*-value	mean fold-change (CC/F)	SEM
K01912	phenylacetate-CoA ligase	Firmicutes	1.0E-155	2.09	0.39
K10823	oligopeptide transport system ATP-binding protein	Firmicutes	7.8E-308	2.08	0.21
K02961	small subunit ribosomal protein S17	Firmicutes	1.1E-03	2.08	0.45
K00965	UDPglucose--hexose-1-phosphate uridylyltransferase	Firmicutes	7.8E-308	2.08	0.03
K07444	putative N6-adenine-specific DNA methylase	Firmicutes	3.9E-114	2.07	0.37
K00942	guanylate kinase	Firmicutes	9.2E-144	2.07	0.39
K00058	D-3-phosphoglycerate dehydrogenase	Firmicutes	3.0E-24	2.07	0.47
K00145	N-acetyl-gamma-glutamyl-phosphate reductase	Firmicutes	1.7E-64	2.07	0.39
K02836	peptide chain release factor 2	Firmicutes	2.6E-171	2.07	0.33
K00926	carbamate kinase	Firmicutes	1.5E-298	2.06	0.19
K03216	tRNA (cytidine/uridine-2'-O-)-methyltransferase	Firmicutes	3.5E-31	2.06	0.40
K03110	fused signal recognition particle receptor	Firmicutes	1.9E-83	2.06	0.40
K06283	putative DeoR family transcriptional regulator, stage III sporulation protein D	Firmicutes	1.1E-10	2.05	0.44
K00995	CDP-diacylglycerol--glycerol-3-phosphate 3-phosphatidyltransferase	Firmicutes	2.3E-119	2.05	0.38
K03786	3-dehydroquinate dehydratase II	Firmicutes	7.8E-308	2.05	0.12
K02878	large subunit ribosomal protein L16	Firmicutes	1.3E-86	2.05	0.37
K00651	homoserine O-succinyltransferase	Firmicutes	2.4E-304	2.05	0.23
K06168	tRNA-2-methylthio-N6-dimethylallyladenosine synthase	Firmicutes	5.4E-04	2.04	0.42
K00029	malate dehydrogenase (oxaloacetate-decarboxylating)(NADP+)	Firmicutes	2.7E-134	2.04	0.18
K11072	spermidine/putrescine transport system ATP-binding protein	Firmicutes	1.1E-287	2.04	0.24
K00789	S-adenosylmethionine synthetase	Firmicutes	6.7E-37	2.04	0.39
K00764	amidophosphoribosyltransferase	Firmicutes	1.5E-112	2.03	0.40
K01187	alpha-glucosidase	Firmicutes	6.9E-21	2.03	0.49
K00821	acetylornithine/N-succinyldiaminopimelate aminotransferase	Firmicutes	2.8E-51	2.03	0.45
K01915	glutamine synthetase	Firmicutes	3.8E-148	2.03	0.37
K01703	3-isopropylmalate/(R)-2-methylmalate dehydratase large subunit	Firmicutes	7.8E-308	2.03	0.05
K03563	carbon storage regulator	Firmicutes	1.5E-26	2.02	0.47

KEGG number	function	phylum	adjusted *p*-value	mean fold-change (CC/F)	SEM
K09157	uncharacterized protein	Firmicutes	7.8E-308	2.01	0.13
K02864	large subunit ribosomal protein L10	Firmicutes	1.2E-24	2.01	0.43
K00041	tagaturonate reductase	Firmicutes	1.6E-290	2.01	0.12
K01889	phenylalanyl-tRNA synthetase alpha chain	Firmicutes	1.3E-108	2.01	0.35
K18344	two-component system, OmpR family, response regulator VanR	Firmicutes	4.5E-145	2.01	0.35
K02871	large subunit ribosomal protein L13	Firmicutes	3.7E-161	2.01	0.31
K01885	glutamyl-tRNA synthetase	Firmicutes	2.7E-53	2.00	0.37
K01791	UDP-N-acetylglucosamine 2-epimerase (non-hydrolysing)	Actinobacteria	9.3E-20	2.00	0.29
K03798	cell division protease FtsH	Streptophyta	6.2E-56	2.00	1.61
K01809	mannose-6-phosphate isomerase	Actinobacteria	3.2E-200	2.00	0.29
K02039	phosphate transport system protein	Firmicutes	1.0E-199	2.00	0.29
K04477	putative hydrolase	Proteobacteria	3.3E-201	2.00	0.29
K05876	methyl-accepting chemotaxis protein III, ribose and galactose sensor receptor	Firmicutes	1.0E-200	2.00	0.29
K06607	myo-inositol catabolism protein IolS	Firmicutes	3.3E-202	2.00	0.29
K11085	ATP-binding cassette, subfamily B, bacterial MsbA	Spirochaetes	1.0E-201	2.00	0.29
K19265	L-glyceraldehyde 3-phosphate reductase	Firmicutes	4.1E-93	2.00	0.37
K13059	N-acetylhexosamine 1-kinase	Firmicutes	2.3E-191	2.00	0.23
K18430	N,N'-diacetyllegionaminate synthase	Firmicutes	1.2E-22	2.00	0.39
K01733	threonine synthase	Firmicutes	6.7E-272	1.99	0.15
K00945	cytidylate kinase	Firmicutes	7.8E-308	1.98	0.16
K00930	acetylglutamate kinase	Firmicutes	6.4E-40	1.97	0.43
K00606	3-methyl-2-oxobutanoate hydroxymethyltransferase	Firmicutes	6.5E-48	1.97	0.26
K01914	aspartate--ammonia ligase	Firmicutes	4.8E-263	1.96	0.25
K01937	CTP synthase	Firmicutes	3.6E-02	1.96	0.39
K04079	molecular chaperone HtpG	Firmicutes	2.0E-65	1.96	0.36
K00873	pyruvate kinase	Firmicutes	7.8E-308	1.95	0.13
K06209	chorismate mutase	Firmicutes	2.8E-180	1.95	0.26
K00928	aspartate kinase	Firmicutes	2.2E-66	1.95	0.35
K19271	chloramphenicol O-acetyltransferase type A	Firmicutes	1.2E-02	1.94	1.60
K00757	uridine phosphorylase	Firmicutes	8.2E-276	1.94	0.09
K01251	adenosylhomocysteinase	Firmicutes	5.3E-10	1.94	0.45
K15534	beta-D-galactosyl-(1->4)-L-rhamnose phosphorylase	Firmicutes	1.1E-204	1.93	0.26
K06405	stage V sporulation protein AC	Firmicutes	1.8E-09	1.93	0.40
K03431	phosphoglucosamine mutase	Firmicutes	1.4E-184	1.93	0.27

KEGG number	function	phylum	adjusted *p*-value	mean fold-change (CC/F)	SEM
K02377	GDP-L-fucose synthase	Firmicutes	5.5E-42	1.92	0.38
K06888	uncharacterized protein	Firmicutes	7.8E-308	1.91	0.37
K01845	glutamate-1-semialdehyde 2,1-aminomutase	Proteobacteria	1.6E-03	1.91	0.31
K01933	phosphoribosylformylglycinamidine cyclo-ligase	Firmicutes	1.2E-55	1.91	0.38
K02952	small subunit ribosomal protein S13	Firmicutes	2.4E-08	1.90	0.39
K01736	chorismate synthase	Firmicutes	6.9E-15	1.90	0.35
K07491	putative transposase	Firmicutes	1.2E-23	1.90	0.36
K03657	DNA helicase II / ATP-dependent DNA helicase PcrA	Firmicutes	6.4E-63	1.90	0.36
K01534	Cd2+/Zn2+-exporting ATPase	Firmicutes	1.1E-35	1.90	0.33
K02890	large subunit ribosomal protein L22	Firmicutes	3.7E-36	1.89	0.34
K03496	chromosome partitioning protein	Firmicutes	9.9E-210	1.89	0.24
K09779	uncharacterized protein	Firmicutes	7.8E-308	1.89	0.32
K06346	spoIIIJ-associated protein	Firmicutes	1.9E-02	1.86	0.40
K01681	aconitate hydratase	Firmicutes	2.6E-49	1.86	0.35
K01945	phosphoribosylamine--glycine ligase	Firmicutes	3.3E-253	1.86	0.22
K04077	chaperonin GroEL	Firmicutes	1.8E-18	1.85	0.33
K03308	neurotransmitter:Na+ symporter, NSS family	Firmicutes	3.0E-42	1.85	0.33
K07406	alpha-galactosidase	Firmicutes	1.1E-40	1.85	0.33
K07738	transcriptional repressor NrdR	Firmicutes	7.8E-308	1.84	0.16
K01738	cysteine synthase A	Firmicutes	1.2E-31	1.84	0.37
K12574	ribonuclease J	Firmicutes	2.8E-02	1.84	0.34
K07669	two-component system, OmpR family, response regulator MprA	Firmicutes	4.4E-21	1.83	0.25
K15912	UDP-N-acetyl-D-glucosamine 4,6-dehydratase	Firmicutes	7.7E-09	1.83	1.59
K09689	capsular polysaccharide transport system ATP-binding protein	Firmicutes	2.6E-101	1.83	0.33
K19789	DNA repair protein RadD	Firmicutes	1.1E-101	1.83	0.33
K15635	2,3-bisphosphoglycerate-independent phosphoglycerate mutase	Firmicutes	7.9E-165	1.83	0.17
K00864	glycerol kinase	Firmicutes	7.6E-13	1.83	0.36
K00067	dTDP-4-dehydrorhamnose reductase	Firmicutes	1.8E-153	1.83	0.19
K03703	excinuclease ABC subunit C	Firmicutes	2.7E-98	1.82	0.29
K03686	molecular chaperone DnaJ	Firmicutes	5.3E-13	1.82	0.32
K01478	arginine deiminase	Firmicutes	1.6E-33	1.82	0.36
K07462	single-stranded-DNA-specific exonuclease	Firmicutes	3.0E-22	1.81	0.24
K02031	peptide/nickel transport system ATP-binding protein	Firmicutes	3.4E-173	1.81	0.26

KEGG number	function	phylum	adjusted *p*-value	mean fold-change (CC/F)	SEM
K02959	small subunit ribosomal protein S16	Firmicutes	2.2E-232	1.81	0.23
K02838	ribosome recycling factor	Firmicutes	1.9E-107	1.80	0.29
K06183	16S rRNA pseudouridine516 synthase	Firmicutes	2.6E-05	1.80	0.37
K15533	1,3-beta-galactosyl-N-acetylhexosamine phosphorylase	Firmicutes	1.1E-182	1.80	0.25
K01929	UDP-N-acetylmuramoyl-tripeptide--D-alanyl-D-alanine ligase	Firmicutes	6.6E-86	1.80	0.29
K02045	sulfate transport system ATP-binding protein	Firmicutes	4.5E-102	1.79	0.18
K11749	regulator of sigma E protease	Firmicutes	1.8E-34	1.78	0.33
K08963	methylthioribose-1-phosphate isomerase	Firmicutes	4.4E-47	1.77	0.32
K02118	V/A-type H+/Na+-transporting ATPase subunit B	Firmicutes	7.8E-308	1.77	0.14
K00812	aspartate aminotransferase	Firmicutes	1.0E-147	1.76	0.21
K02956	small subunit ribosomal protein S15	Firmicutes	2.9E-86	1.75	0.29
K10539	L-arabinose transport system ATP-binding protein	Firmicutes	3.0E-14	1.75	0.25
K03550	holliday junction DNA helicase RuvA	Firmicutes	1.1E-237	1.75	0.18
K11754	dihydrofolate synthase / folylpolyglutamate synthase	Firmicutes	5.7E-80	1.74	0.24
K01918	pantoate--beta-alanine ligase	Firmicutes	4.8E-88	1.74	0.26
K01958	pyruvate carboxylase	Firmicutes	1.5E-72	1.73	0.19
K06958	RNase adapter protein RapZ	Firmicutes	1.4E-08	1.73	0.33
K03617	electron transport complex protein RnfA	Firmicutes	7.5E-22	1.72	0.29
K03665	GTPase	Firmicutes	2.0E-249	1.72	0.17
K02892	large subunit ribosomal protein L23	Firmicutes	3.0E-265	1.71	0.13
K02916	large subunit ribosomal protein L35	Firmicutes	1.9E-133	1.70	0.22
K00128	aldehyde dehydrogenase (NAD+)	Firmicutes	9.5E-93	1.70	0.20
K08602	oligoendopeptidase F	Firmicutes	2.7E-106	1.70	0.25
K01854	UDP-galactopyranose mutase	Firmicutes	1.7E-305	1.70	0.14
K00099	1-deoxy-D-xylulose-5-phosphate reductoisomerase	Firmicutes	2.0E-136	1.68	0.21
K00858	NAD+ kinase	Firmicutes	7.5E-179	1.68	0.19
K05601	hydroxylamine reductase	Firmicutes	2.8E-137	1.68	0.19
K07651	two-component system, OmpR family, sensor histidine kinase ResE	Firmicutes	5.1E-92	1.68	0.19
K01783	ribulose-phosphate 3-epimerase	Firmicutes	4.2E-11	1.68	0.31
K01870	isoleucyl-tRNA synthetase	Firmicutes	9.3E-04	1.67	0.27
K10010	cystine transport system ATP-binding protein	Firmicutes	1.2E-67	1.67	0.18
K11751	5'-nucleotidase / UDP-sugar diphosphatase	Firmicutes	1.7E-04	1.67	1.36

KEGG number	function	phylum	adjusted *p*-value	mean fold-change (CC/F)	SEM
K03336	3D-(3,5/4)-trihydroxycyclohexane-1,2-dione acylhydrolase (decyclizing)	Firmicutes	2.0E-96	1.66	0.23
K00265	glutamate synthase (NADPH/NADH) large chain	Firmicutes	9.6E-49	1.66	0.25
K15582	oligopeptide transport system permease protein	Firmicutes	3.3E-19	1.65	0.24
K02028	polar amino acid transport system ATP-binding protein	Firmicutes	6.4E-36	1.62	0.24
K01995	branched-chain amino acid transport system ATP-binding protein	Firmicutes	6.6E-26	1.62	0.24
K03498	trk system potassium uptake protein	Firmicutes	1.8E-48	1.61	0.21
K01486	adenine deaminase	Firmicutes	2.5E-20	1.61	0.24
K02117	V/A-type H+/Na+-transporting ATPase subunit A	Firmicutes	4.0E-283	1.60	0.13
K03525	type III pantothenate kinase	Firmicutes	1.1E-02	1.57	0.26
K06997	PLP dependent protein	Firmicutes	7.0E-54	1.57	0.16
K00027	malate dehydrogenase (oxaloacetate-decarboxylating)	Firmicutes	3.4E-246	1.56	0.13
K02010	iron(III) transport system ATP-binding protein	Firmicutes	1.4E-43	1.55	0.07
K02032	peptide/nickel transport system ATP-binding protein	Firmicutes	3.4E-173	1.55	0.19
K01267	aspartyl aminopeptidase	Firmicutes	7.8E-44	1.54	0.17
K01897	long-chain acyl-CoA synthetase	Firmicutes	5.5E-34	1.53	0.15
K00919	4-diphosphocytidyl-2-C-methyl-D-erythritol kinase	Firmicutes	9.3E-181	1.48	0.10
K07133	uncharacterized protein	Firmicutes	7.8E-308	1.24	1.12
K10206	LL-diaminopimelate aminotransferase	Firmicutes	3.8E-135	1.20	0.05
K03321	sulfate permease, SulP family	Firmicutes	8.3E-47	1.17	1.10
K00014	shikimate dehydrogenase	Bacteroidetes	1.2E-38	-1.51	0.20
K03183	demethylmenaquinone methyltransferase / 2-methoxy-6-polyprenyl-1,4-benzoquinol methylase	Bacteroidetes	4.8E-24	-1.57	0.11
K09760	DNA recombination protein RmuC	Bacteroidetes	1.1E-87	-1.59	0.20
K02426	cysteine desulfuration protein SufE	Bacteroidetes	1.8E-149	-1.60	0.07
K03768	peptidyl-prolyl cis-trans isomerase B (cyclophilin B)	Bacteroidetes	5.3E-06	-1.61	0.24
K00763	nicotinate phosphoribosyltransferase	Bacteroidetes	2.9E-124	-1.62	0.19
K04773	protease IV	Bacteroidetes	3.4E-35	-1.63	0.24
K01854	UDP-galactopyranose mutase	Bacteroidetes	2.9E-28	-1.63	0.24
K00764	amidophosphoribosyltransferase	Bacteroidetes	9.7E-10	-1.67	0.17
K03525	type III pantothenate kinase	Bacteroidetes	6.0E-113	-1.68	0.13

KEGG number	function	phylum	adjusted *p*-value	mean fold-change (CC/F)	SEM
K00282	glycine dehydrogenase subunit 1	Bacteroidetes	3.8E-13	-1.73	0.28
K02495	oxygen-independent coproporphyrinogen III oxidase	Bacteroidetes	3.1E-63	-1.75	0.40
K02013	iron complex transport system ATP-binding protein	Bacteroidetes	3.3E-74	-1.75	0.25
K03442	small conductance mechanosensitive channel	Bacteroidetes	3.4E-48	-1.76	0.27
K02066	phospholipid/cholesterol/gamma-HCH transport system permease protein	Bacteroidetes	5.4E-136	-1.78	0.27
K13683	putative colanic acid biosynthesis glycosyltransferase	Bacteroidetes	3.1E-77	-1.78	0.26
K08169	MFS transporter, DHA2 family, multidrug resistance protein	Proteobacteria	1.9E-03	-1.78	0.28
K09951	CRISPR-associated protein Cas2	Bacteroidetes	6.9E-159	-1.80	0.03
K05837	rod shape determining protein RodA	Bacteroidetes	4.5E-25	-1.81	0.31
K07715	two-component system, NtrC family, response regulator GlrR	Bacteroidetes	3.9E-10	-1.82	0.36
K00605	aminomethyltransferase	Proteobacteria	2.0E-99	-1.83	0.33
K00864	glycerol kinase	Tenericutes	1.1E-98	-1.83	0.33
K01491	methylenetetrahydrofolate dehydrogenase (NADP+) / methenyltetrahydrofolate cyclohydrolase	Euryarchaeota	3.5E-100	-1.83	0.33
K02006	cobalt/nickel transport system ATP-binding protein	Euryarchaeota	1.5E-100	-1.83	0.33
K03702	excinuclease ABC subunit B	Fusobacteria	8.4E-100	-1.83	0.33
K18673	beta-glucoside kinase	Firmicutes	4.7E-99	-1.83	0.33
K01760	cystathionine beta-lyase	Bacteroidetes	7.9E-03	-1.83	0.17
K05540	tRNA-dihydrouridine synthase B	Bacteroidetes	9.2E-89	-1.83	0.28
K06889	uncharacterized protein	Bacteroidetes	1.1E-306	-1.86	0.30
K17828	dihydroorotate dehydrogenase (NAD+) catalytic subunit	Bacteroidetes	7.8E-308	-1.86	0.22
K03101	signal peptidase II	Bacteroidetes	3.1E-270	-1.89	0.22
K00046	gluconate 5-dehydrogenase	Bacteroidetes	2.2E-170	-1.90	0.22
K04078	chaperonin GroES	Bacteroidetes	1.8E-256	-1.91	0.29
K01462	peptide deformylase	Bacteroidetes	1.2E-39	-1.91	0.35
K07139	uncharacterized protein	Bacteroidetes	1.1E-306	-1.94	0.34
K03839	flavodoxin I	Bacteroidetes	5.4E-62	-1.95	0.26
K00929	butyrate kinase	Bacteroidetes	2.0E-06	-1.96	0.44
K01696	tryptophan synthase beta chain	Bacteroidetes	5.1E-223	-1.96	0.15
K02601	transcriptional antiterminator NusG	Bacteroidetes	4.3E-03	-1.97	0.51
K03387	alkyl hydroperoxide reductase subunit F	Bacteroidetes	2.5E-87	-1.98	0.38

KEGG number	function	phylum	adjusted *p*-value	mean fold-change (CC/F)	SEM
K07085	putative transport protein	Proteobacteria	1.1E-05	-1.98	0.40
K02429	MFS transporter, FHS family, L-fucose permease	Bacteroidetes	1.5E-16	-1.99	0.46
K00783	23S rRNA (pseudouridine1915-N3)-methyltransferase	Bacteroidetes	2.2E-16	-2.00	0.39
K03502	DNA polymerase V	Proteobacteria	3.1E-198	-2.00	0.29
K03546	exonuclease SbcC	Proteobacteria	9.8E-199	-2.00	0.29
K03723	transcription-repair coupling factor (superfamily II helicase)	Proteobacteria	7.7E-19	-2.00	0.29
K02316	DNA primase	Proteobacteria	3.1E-34	-2.00	0.00
K02518	translation initiation factor IF-1	Bacteroidetes	1.0E-08	-2.01	0.39
K18118	succinyl-CoA:acetate CoA-transferase	Firmicutes	2.9E-21	-2.03	0.32
K00800	3-phosphoshikimate 1-carboxyvinyltransferase	Bacteroidetes	6.8E-38	-2.03	0.47
K00548	5-methyltetrahydrofolate--homocysteine methyltransferase	Proteobacteria	1.6E-286	-2.04	0.32
K03210	preprotein translocase subunit YajC	Bacteroidetes	2.8E-25	-2.05	0.43
K03781	catalase	Proteobacteria	9.4E-34	-2.06	0.51
K03701	excinuclease ABC subunit A	Fusobacteria	4.0E-05	-2.08	0.22
K01662	1-deoxy-D-xylulose-5-phosphate synthase	Fusobacteria	1.2E-37	-2.10	0.38
K07304	peptide-methionine (S)-S-oxide reductase	Bacteroidetes	1.9E-301	-2.11	0.14
K01766	cysteine sulfinate desulfinase	Bacteroidetes	5.1E-273	-2.11	0.28
K01267	aspartyl aminopeptidase	Bacteroidetes	1.9E-22	-2.14	0.47
K02916	large subunit ribosomal protein L35	Bacteroidetes	8.3E-33	-2.15	0.44
K07238	zinc transporter, ZIP family	Firmicutes	1.7E-130	-2.15	0.15
K00610	aspartate carbamoyltransferase regulatory subunit	Bacteroidetes	7.8E-308	-2.16	0.05
K15915	undecaprenyl phosphate N,N'-diacetylbacillosamine 1-phosphate transferase	Bacteroidetes	1.7E-250	-2.17	0.33
K01585	arginine decarboxylase	Proteobacteria	2.1E-261	-2.17	0.17
K03386	peroxiredoxin (alkyl hydroperoxide reductase subunit C)	Bacteroidetes	6.3E-91	-2.17	0.42
K00609	aspartate carbamoyltransferase catalytic subunit	Actinobacteria	3.8E-84	-2.19	0.43
K01338	ATP-dependent Lon protease	Bacteroidetes	5.3E-51	-2.21	0.46
K00873	pyruvate kinase	Bacteroidetes	6.4E-170	-2.21	0.38
K04485	DNA repair protein RadA/Sms	Bacteroidetes	7.8E-308	-2.22	0.22
K01258	tripeptide aminopeptidase	Proteobacteria	7.1E-75	-2.22	0.28
K01662	1-deoxy-D-xylulose-5-phosphate synthase	Bacteroidetes	2.2E-15	-2.23	0.53

KEGG number	function	phylum	adjusted *p*-value	mean fold-change (CC/F)	SEM
K01265	methionyl aminopeptidase	Actinobacteria	1.8E-54	-2.23	0.15
K10912	two-component system, repressor protein LuxO	Bacteroidetes	1.2E-80	-2.25	0.14
K02564	glucosamine-6-phosphate deaminase	Bacteroidetes	3.4E-238	-2.26	0.37
K01577	oxalyl-CoA decarboxylase	Actinobacteria	5.5E-104	-2.27	0.15
K03585	membrane fusion protein, multidrug efflux system	Bacteroidetes	6.6E-178	-2.27	0.25
K01952	phosphoribosylformylglycinamidine synthase	Bacteroidetes	8.4E-289	-2.28	0.32
K01414	oligopeptidase A	Proteobacteria	6.8E-03	-2.28	0.86
K01414	oligopeptidase A	Bacteroidetes	1.3E-27	-2.30	0.48
K11991	tRNA(adenine34) deaminase	Bacteroidetes	1.4E-70	-2.31	0.50
K01425	glutaminase	Bacteroidetes	7.8E-308	-2.31	0.30
K00041	tagaturonate reductase	Bacteroidetes	2.8E-165	-2.31	0.49
K18197	rhamnogalacturonan endolyase	Bacteroidetes	3.9E-152	-2.32	0.46
K04043	molecular chaperone DnaK	Cyanobacteria	4.3E-287	-2.33	0.44
K07305	peptide-methionine (R)-S-oxide reductase	Proteobacteria	5.4E-163	-2.33	0.44
K06201	copper homeostasis protein	Spirochaetes	2.5E-04	-2.33	0.73
K03565	regulatory protein	Firmicutes	3.2E-94	-2.33	0.17
K03070	preprotein translocase subunit SecA	Proteobacteria	9.3E-55	-2.34	0.66
K00641	homoserine O-acetyltransferase	Bacteroidetes	9.4E-132	-2.38	0.55
K08138	MFS transporter, SP family, xylose:H+ symportor	Bacteroidetes	2.7E-254	-2.41	0.44
K00633	galactoside O-acetyltransferase	Bacteroidetes	2.9E-46	-2.41	0.69
K07387	putative metalloprotease	Bacteroidetes	4.5E-04	-2.41	0.65
K11737	D-serine/D-alanine/glycine transporter	Firmicutes	2.7E-140	-2.43	0.23
K00680	[no name]	Bacteroidetes	3.9E-237	-2.43	0.29
K01580	glutamate decarboxylase	Bacteroidetes	1.0E-206	-2.44	0.42
K05349	beta-glucosidase	Proteobacteria	3.0E-78	-2.44	0.63
K06959	protein Tex	Proteobacteria	5.3E-187	-2.51	0.36
K01520	dUTP pyrophosphatase	Fusobacteria	1.4E-02	-2.52	0.87
K11754	dihydrofolate synthase / folylpolyglutamate synthase	Bacteroidetes	8.0E-30	-2.54	0.60
K03466	DNA segregation ATPase FtsK/SpoIIIE, S-DNA-T family	Proteobacteria	2.2E-11	-2.56	0.87
K00805	heptaprenyl diphosphate synthase	Bacteroidetes	1.5E-75	-2.56	0.73
K03732	ATP-dependent RNA helicase RhlB	Bacteroidetes	2.5E-126	-2.56	0.65
K03310	alanine or glycine:cation symporter, AGCS family	Bacteroidetes	7.8E-308	-2.58	0.17
K01356	repressor LexA	Bacteroidetes	1.1E-151	-2.58	0.25
K00076	7-alpha-hydroxysteroid dehydrogenase	Bacteroidetes	8.5E-266	-2.58	0.55

KEGG number	function	phylum	adjusted *p*-value	mean fold-change (CC/F)	SEM
K09817	zinc transport system ATP-binding protein	Bacteroidetes	3.9E-274	-2.59	0.30
K00651	homoserine O-succinyltransferase	Bacteroidetes	6.4E-220	-2.60	0.84
K01207	beta-N-acetylhexosaminidase	Bacteroidetes	9.2E-120	-2.61	0.50
K03976	Cys-tRNA(Pro)/Cys-tRNA(Cys) deacylase	Bacteroidetes	4.6E-02	-2.61	0.88
K01685	altronate hydrolase	Bacteroidetes	3.4E-144	-2.63	0.66
K07749	formyl-CoA transferase	Actinobacteria	7.8E-308	-2.64	0.22
K10907	aminotransferase	Bacteroidetes	1.0E-299	-2.65	0.37
K05837	rod shape determining protein RodA	Firmicutes	7.8E-308	-2.67	0.73
K02529	LacI family transcriptional regulator	Bacteroidetes	2.0E-158	-2.67	0.73
K07271	lipopolysaccharide cholinephosphotransferase	Bacteroidetes	7.2E-134	-2.67	0.67
K03737	pyruvate-ferredoxin/flavodoxin oxidoreductase	Proteobacteria	4.1E-65	-2.70	0.48
K03614	electron transport complex protein RnfD	Bacteroidetes	3.6E-40	-2.70	0.85
K18197	rhamnogalacturonan endolyase	Firmicutes	1.9E-272	-2.72	0.15
K03702	excinuclease ABC subunit B	Actinobacteria	7.6E-124	-2.73	1.05
K02377	GDP-L-fucose synthase	Bacteroidetes	1.6E-02	-2.76	0.77
K01738	cysteine synthase A	Actinobacteria	2.4E-266	-2.78	0.36
K10773	endonuclease III	Proteobacteria	8.5E-122	-2.78	0.62
K10206	LL-diaminopimelate aminotransferase	Spirochaetes	1.9E-188	-2.83	1.09
K01498	diaminohydroxyphosphoribosylamino pyrimidine deaminase	Bacteroidetes	3.9E-105	-2.83	1.09
K03503	DNA polymerase V	Bacteroidetes	1.7E-25	-2.84	0.88
K09811	cell division transport system permease protein	Bacteroidetes	1.5E-97	-2.87	0.98
K15987	K(+)-stimulated pyrophosphate-energized sodium pump	Bacteroidetes	9.2E-03	-2.87	0.87
K02114	F-type H+-transporting ATPase subunit epsilon	Bacteroidetes	4.6E-96	-2.89	0.95
K01897	long-chain acyl-CoA synthetase	Proteobacteria	7.8E-308	-2.89	0.11
K00789	S-adenosylmethionine synthetase	Actinobacteria	5.0E-235	-2.92	1.06
K18138	multidrug efflux pump	Proteobacteria	1.2E-245	-2.93	0.46
K14155	cystathione beta-lyase	Bacteroidetes	2.2E-241	-2.94	0.69
K01754	threonine dehydratase	Bacteroidetes	7.8E-308	-2.97	0.51
K01950	NAD+ synthase (glutamine-hydrolysing)	Actinobacteria	2.7E-214	-2.97	0.39
K01879	glycyl-tRNA synthetase beta chain	Firmicutes	1.2E-21	-2.98	1.15
K05592	ATP-dependent RNA helicase DeaD	Actinobacteria	7.8E-308	-3.00	1.04
K02017	molybdate transport system ATP-binding protein	Bacteroidetes	3.1E-181	-3.00	1.00

KEGG number	function	phylum	adjusted *p*-value	mean fold-change (CC/F)	SEM
K01894	glutamyl-Q tRNA(Asp) synthetase	Actinobacteria	7.5E-171	-3.00	0.76
K11626	putative sodium/glutamine symporter	Bacteroidetes	6.4E-305	-3.01	0.58
K15022	formate dehydrogenase beta subunit	Bacteroidetes	3.1E-292	-3.02	0.82
K01693	imidazoleglycerol-phosphate dehydratase	Proteobacteria	7.7E-255	-3.10	0.56
K07118	uncharacterized protein	Bacteroidetes	1.1E-306	-3.15	0.08
K02470	DNA gyrase subunit B	Actinobacteria	1.6E-223	-3.16	0.50
K00721	dolichol-phosphate mannosyltransferase	Actinobacteria	7.8E-308	-3.17	0.83
K12506	2-C-methyl-D-erythritol 4-phosphate cytidylyltransferase / 2-C-methyl-D-erythritol 2,4-cyclodiphosphate synthase	Proteobacteria	2.1E-04	-3.18	1.03
K03781	catalase	Bacteroidetes	5.4E-139	-3.21	0.89
K00991	2-C-methyl-D-erythritol 4-phosphate cytidylyltransferase	Bacteroidetes	1.8E-221	-3.21	0.69
K02316	DNA primase	Actinobacteria	7.8E-308	-3.21	0.98
K00954	pantetheine-phosphate adenylyltransferase	Proteobacteria	1.1E-264	-3.22	0.89
K00384	thioredoxin reductase (NADPH)	Actinobacteria	4.5E-175	-3.26	0.87
K12952	cation-transporting ATPase E	Actinobacteria	7.8E-308	-3.33	1.36
K01220	6-phospho-beta-galactosidase	Firmicutes	1.0E-160	-3.33	1.64
K01281	X-Pro dipeptidyl-peptidase	Firmicutes	4.1E-258	-3.39	0.87
K02006	cobalt/nickel transport system ATP-binding protein	Proteobacteria	3.0E-275	-3.43	1.02
K07088	uncharacterized protein	Firmicutes	7.8E-308	-3.44	0.73
K03088	RNA polymerase sigma-70 factor, ECF subfamily	Bacteroidetes	1.0E-172	-3.48	1.21
K03549	KUP system potassium uptake protein	Firmicutes	7.8E-308	-3.48	0.29
K15726	cobalt-zinc-cadmium resistance protein CzcA	Bacteroidetes	5.7E-234	-3.50	1.32
K07386	putative endopeptidase	Bacteroidetes	3.3E-41	-3.53	1.54
K13683	putative colanic acid biosynthesis glycosyltransferase	Proteobacteria	3.2E-76	-3.55	1.77
K03606	putative colanic acid biosysnthesis UDP-glucose lipid carrier transferase	Bacteroidetes	5.2E-109	-3.55	1.02
K00567	methylated-DNA-[protein]-cysteine S-methyltransferase	Bacteroidetes	7.8E-308	-3.63	1.08
K01876	aspartyl-tRNA synthetase	Proteobacteria	1.3E-58	-3.65	1.59
K02193	heme exporter protein A	Bacteroidetes	1.4E-19	-3.67	2.19
K03596	GTP-binding protein LepA	Actinobacteria	1.9E-267	-3.71	1.55
K02892	large subunit ribosomal protein L23	Bacteroidetes	1.5E-63	-3.72	1.54
K00024	malate dehydrogenase	Firmicutes	5.0E-149	-3.78	2.11
K04020	phosphotransacetylase	Bacteroidetes	7.8E-308	-3.83	0.83

**KEGG number**	**function**	**phylum**	**adjusted *p*-value**	**mean fold-change (CC/F)**	**SEM**
K08998	uncharacterized protein	Actinobacteria	7.2E-218	-3.83	2.13
K03424	TatD DNase family protein	Bacteroidetes	4.7E-55	-3.87	1.58
K01885	glutamyl-tRNA synthetase	Actinobacteria	3.2E-72	-3.88	1.99
K19005	lipoteichoic acid synthase	Firmicutes	2.9E-284	-3.94	1.28
K07137	uncharacterized protein	Bacteroidetes	1.1E-306	-4.18	1.88
K03615	electron transport complex protein RnfC	Proteobacteria	8.7E-271	-4.25	1.66
K02950	small subunit ribosomal protein S12	Proteobacteria	2.8E-69	-4.41	2.55
K00265	glutamate synthase (NADPH/NADH) large chain	Actinobacteria	2.6E-68	-4.71	2.76
K00611	ornithine carbamoyltransferase	Actinobacteria	2.7E-194	-4.78	2.99
K18940	two-component system, OmpR family, sensor histidine kinase ArlS	Bacteroidetes	7.5E-07	-4.85	3.58
K04096	DNA processing protein	Bacteroidetes	3.1E-71	-4.92	3.07
K12267	peptide methionine sulfoxide reductase msrA/msrB	Firmicutes	7.8E-308	-5.00	1.53
K03744	LemA protein	Proteobacteria	1.3E-278	-5.08	2.97
K00858	NAD+ kinase	Proteobacteria	3.2E-209	-5.17	2.46
K02469	DNA gyrase subunit A	Actinobacteria	7.8E-308	-5.42	2.68
K13954	alcohol dehydrogenase	Bacteroidetes	1.4E-224	-5.56	3.31
K03499	trk system potassium uptake protein	Bacteroidetes	7.8E-308	-5.60	3.21
K00603	glutamate formiminotransferase	Bacteroidetes	9.4E-254	-5.74	2.39
K03549	KUP system potassium uptake protein	Actinobacteria	7.8E-308	-5.81	2.47

Table S2. Functional-taxonomic features with significantly differential abundance between cecal and fecal metaproteome.

KEGG number	function	phylum	adjusted *p*-value	mean fold-change (CC/F)	SEM
K03406	methyl-accepting chemotaxis protein	Firmicutes	2.70E-33	8.36	5.41
K02012	iron(III) transport system substrate-binding protein	Firmicutes	4.30E-53	6.92	4.8
K01923	phosphoribosylaminoimidazole-succinocarboxamide synthase	Firmicutes	4.40E-37	5.24	2.75
K02078	acyl carrier protein	Firmicutes	3.20E-60	5.06	1.7
K01358	ATP-dependent Clp protease, protease subunit	Firmicutes	5.20E-55	4.72	2.01
K19709	acetate CoA-transferase	Firmicutes	7.70E-52	4.44	1.82
K01876	aspartyl-tRNA synthetase	Firmicutes	4.00E-21	4.34	2.59
K03336	3D-(3,5/4)-trihydroxycyclohexane-1,2-dione acylhydrolase (decyclizing)	Firmicutes	2.20E-19	4.31	2.23
K01835	phosphoglucomutase	Firmicutes	9.40E-39	4.27	1.05
K00962	polyribonucleotide nucleotidyltransferase	Firmicutes	6.90E-61	4.27	0.29
K01810	glucose-6-phosphate isomerase	Firmicutes	7.30E-03	4.03	1.74
K00602	phosphoribosylaminoimidazolecarboxamide formyltransferase / IMP cyclohydrolase	Firmicutes	9.10E-33	4.03	1.82
K00616	transaldolase	Firmicutes	4.60E-07	3.95	2.02
K02014	iron complex outermembrane recepter protein	Ascomycota	1.70E-16	3.5	1.8
K04047	starvation-inducible DNA-binding protein	Thermodesulfobacteria	3.30E-31	3.39	1.06
K10540	methyl-galactoside transport system substrate-binding protein	Firmicutes	1.60E-44	3.38	0.5
K00656	formate C-acetyltransferase	Firmicutes	1.10E-10	3.36	1.26
K00010	myo-inositol 2-dehydrogenase / D-chiro-inositol 1-dehydrogenase	Firmicutes	1.10E-29	3.07	0.64
K01571	oxaloacetate decarboxylase, alpha subunit	Firmicutes	4.00E-48	3.06	0.24
K01610	phosphoenolpyruvate carboxykinase (ATP)	Firmicutes	6.20E-44	2.95	0.58

KEGG number	function	phylum	adjusted *p*-value	mean fold-change (CC/F)	SEM
K04077	chaperonin GroEL	Firmicutes	1.90E-13	2.91	0.92
K04072	acetaldehyde dehydrogenase / alcohol dehydrogenase	Firmicutes	1.30E-41	2.84	0.6
K00042	2-hydroxy-3-oxopropionate reductase	Firmicutes	1.40E-21	2.83	0.87
K01890	phenylalanyl-tRNA synthetase beta chain	Firmicutes	1.20E-55	2.83	0.17
K02117	V/A-type H+/Na+-transporting ATPase subunit A	Firmicutes	3.00E-20	2.78	0.83
K01966	propionyl-CoA carboxylase beta chain	Firmicutes	8.60E-07	2.55	0.57
K01812	glucuronate isomerase	Firmicutes	1.80E-08	2.41	0.72
K02863	large subunit ribosomal protein L1	Firmicutes	4.10E-45	2.39	0.07
K06972	presequence protease	Firmicutes	7.00E-40	2.33	0.44
K02982	small subunit ribosomal protein S3	Firmicutes	2.50E-15	2.29	0.37
K02357	elongation factor Ts	Firmicutes	8.10E-14	2.25	0.43
K13920	propanediol dehydratase small subunit	Firmicutes	3.30E-03	2.25	0.38
K01667	tryptophanase	Firmicutes	6.70E-08	2.17	0.17
K11072	spermidine/putrescine transport system ATP-binding protein	Firmicutes	7.90E-34	2.17	0.44
K01915	glutamine synthetase	Firmicutes	6.30E-23	2.08	0.08
K00850	6-phosphofructokinase 1	Firmicutes	3.40E-38	2.02	0.23
K01804	L-arabinose isomerase	Firmicutes	1.20E-37	2.02	0.05
K02014	iron complex outermembrane recepter protein	Proteobacteria	3.00E-25	2	0.29
K02931	large subunit ribosomal protein L5	Firmicutes	6.60E-17	1.94	0.42
K00927	phosphoglycerate kinase	Firmicutes	1.00E-45	1.92	0.15
K00975	glucose-1-phosphate adenylyltransferase	Firmicutes	1.10E-30	1.87	0.16
K00262	glutamate dehydrogenase (NADP+)	Firmicutes	1.50E-14	1.79	0.27
K00053	ketol-acid reductoisomerase	Firmicutes	3.50E-42	1.78	0.17
K05795	tellurium resistance protein TerD	Firmicutes	5.10E-22	1.74	0.08
K01255	leucyl aminopeptidase	Firmicutes	1.40E-03	1.67	0.17
K03555	DNA mismatch repair protein MutS	Firmicutes	1.40E-03	1.67	0.17

KEGG number	function	phylum	adjusted *p*-value	mean fold-change (CC/F)	SEM
K02864	large subunit ribosomal protein L10	Firmicutes	3.30E-26	1.59	0.11
K00248	butyryl-CoA dehydrogenase	Firmicutes	2.50E-07	1.57	0.39
K10546	putative multiple sugar transport system substrate-binding protein	Firmicutes	4.40E-09	1.53	0.2
K03641	TolB protein	Bacteroidetes	2.80E-05	-1.65	0.36
K00262	glutamate dehydrogenase (NADP+)	Bacteroidetes	2.40E-43	-1.67	0.11
K01689	enolase	Bacteroidetes	3.30E-51	-1.7	0.12
K00239	succinate dehydrogenase / fumarate reductase, flavoprotein subunit	Bacteroidetes	5.00E-41	-1.78	0.1
K03781	catalase	Bacteroidetes	1.00E-15	-1.83	0.33
K01881	prolyl-tRNA synthetase	Bacteroidetes	9.90E-26	-2	0.29
K01805	xylose isomerase	Bacteroidetes	5.50E-36	-2	0.14
K02878	large subunit ribosomal protein L16	Bacteroidetes	1.10E-02	-2.09	0.56
K03783	purine-nucleoside phosphorylase	Bacteroidetes	5.70E-19	-2.22	0.36
K11175	phosphoribosylglycinamide formyltransferase 1	Bacteroidetes	5.70E-04	-2.23	0.43
K01875	seryl-tRNA synthetase	Bacteroidetes	1.50E-18	-2.28	0.43
K02884	large subunit ribosomal protein L19	Bacteroidetes	3.50E-27	-2.34	0.2
K00849	galactokinase	Bacteroidetes	8.90E-09	-2.39	0.58
K02890	large subunit ribosomal protein L22	Bacteroidetes	5.20E-06	-2.6	0.57
K00024	malate dehydrogenase	Bacteroidetes	1.80E-52	-2.69	0.31
K00912	tetraacyldisaccharide 4'-kinase	Bacteroidetes	1.90E-35	-2.94	0.06
K00874	2-dehydro-3-deoxygluconokinase	Bacteroidetes	9.30E-43	-2.95	0.73
K02014	iron complex outermembrane recepter protein	Bacteroidetes	1.60E-47	-2.97	0.47
K02990	small subunit ribosomal protein S6	Bacteroidetes	1.20E-28	-3	0.66
K00384	thioredoxin reductase (NADPH)	Bacteroidetes	1.50E-59	-3.17	0.73
K00705	4-alpha-glucanotransferase	Bacteroidetes	6.10E-57	-3.67	1.92
K01619	deoxyribose-phosphate aldolase	Bacteroidetes	2.70E-56	-3.83	0.29
K01424	L-asparaginase	Bacteroidetes	3.20E-62	-4.52	0.78
K01270	dipeptidase D	Bacteroidetes	2.60E-24	-4.62	2.21

KEGG number	function	phylum	adjusted *p*-value	mean fold-change (CC/F)	SEM
K02871	large subunit ribosomal protein L13	Bacteroidetes	2.40E-10	-5.16	3.06
K04564	superoxide dismutase, Fe-Mn family	Bacteroidetes	9.70E-49	-5.49	1.93
K03386	peroxiredoxin (alkyl hydroperoxide reductase subunit C)	Bacteroidetes	2.30E-34	-5.62	2.87
K02406	flagellin	Proteobacteria	7.60E-24	-6.17	4.44
K01200	pullulanase	Bacteroidetes	1.40E-63	-9.25	3.7
K04043	molecular chaperone DnaK	Bacteroidetes	6.00E-15	-9.91	4.81
K01425	glutaminase	Bacteroidetes	3.00E-64	-11.39	2.27
K01577	oxalyl-CoA decarboxylase	Bacteroidetes	2.70E-66	-13.72	4.33
K01580	glutamate decarboxylase	Bacteroidetes	1.30E-65	-17.08	6.43
K07749	formyl-CoA transferase	Bacteroidetes	6.30E-65	-22.09	4.62

Table S3. Firmicutes function with significantly differential normalized abundance between cecal and fecal metagenome.

KEGG number	function	adjusted *p*-value	mean fold-change (CC/F)	SEM
K15532	unsaturated rhamnogalacturonyl hydrolase	3.3E-07	5.93	4.05
K01666	4-hydroxy 2-oxovalerate aldolase	2.0E-26	4.47	1.02
K00690	sucrose phosphorylase	1.5E-17	4.44	1.54
K00140	malonate-semialdehyde dehydrogenase (acetylating) / methylmalonate-semialdehyde dehydrogenase	1.0E-18	3.33	1.09
K01598	phosphopantothenoylcysteine decarboxylase	4.1E-27	3.17	0.44
K07402	xanthine dehydrogenase accessory factor	1.5E-03	3.07	1.27
K01611	S-adenosylmethionine decarboxylase	7.7E-21	2.92	0.35
K00209	enoyl-[acyl-carrier protein] reductase / trans-2-enoyl-CoA reductase (NAD+)	7.1E-10	2.83	0.60
K16951	anaerobic sulfite reductase subunit B	8.8E-11	2.67	0.33
K07696	two-component system, NarL family, response regulator NreC	1.0E-24	2.67	0.60
K00872	homoserine kinase	5.1E-07	2.47	0.29
K09684	purine catabolism regulatory protein	5.8E-12	2.44	0.29
K01069	hydroxyacylglutathione hydrolase	8.8E-22	2.33	0.17
K04562	flagellar biosynthesis protein FlhG	3.2E-20	2.33	0.44
K06148	ATP-binding cassette, subfamily C, bacterial	2.7E-06	2.32	0.49
K13731	internalin B	5.7E-17	2.17	0.44
K11923	MerR family transcriptional regulator, copper efflux regulator	3.0E-17	2.17	0.44
K06284	transcriptional pleiotropic regulator of transition state genes	4.0E-05	2.06	0.34
K03778	D-lactate dehydrogenase	2.2E-08	2.04	0.31
K02420	flagellar biosynthetic protein FliQ	8.7E-08	2.04	0.09
K10017	histidine transport system ATP-binding protein	8.0E-03	2.03	0.29
K03476	L-ascorbate 6-phosphate lactonase	4.2E-02	2.01	0.32
K05910	NADH peroxidase	3.3E-12	2.00	0.29
K00111	glycerol-3-phosphate dehydrogenase	1.5E-10	2.00	0.50
K01154	type I restriction enzyme, S subunit	1.9E-12	2.00	0.29
K00957	sulfate adenylyltransferase subunit 2	3.2E-09	1.97	0.23
K10440	ribose transport system permease protein	1.9E-13	1.90	0.17
K07775	two-component system, OmpR family, response regulator ResD	1.3E-19	1.88	0.18
K03783	purine-nucleoside phosphorylase	5.8E-05	1.67	0.20
K02879	large subunit ribosomal protein L17	3.8E-21	1.64	0.11
K06972	presequence protease	2.2E-23	1.62	0.05
K02356	elongation factor P	4.7E-24	1.52	0.02
K13653	AraC family transcriptional regulator	5.7E-14	1.51	0.03
K02763	PTS system, D-glucosamine-specific IIA component	2.3E-04	1.42	0.11
K00640	serine O-acetyltransferase	8.4E-09	1.40	0.07
K02115	F-type H+-transporting ATPase subunit gamma	3.4E-02	1.38	0.13
K02967	small subunit ribosomal protein S2	6.0E-13	1.35	0.09
K02867	large subunit ribosomal protein L11	1.9E-05	1.33	0.11
K02406	flagellin	3.1E-14	1.33	0.07

KEGG number	function	adjusted *p*-value	mean fold-change (CC/F)	SEM
K00973	glucose-1-phosphate thymidylyltransferase	1.4E-08	1.30	0.03
K01923	phosphoribosylaminoimidazole-succinocarboxamide synthase	1.7E-02	1.28	0.10
K01866	tyrosyl-tRNA synthetase	1.1E-16	1.25	0.04
K16786	energy-coupling factor transport system ATP-binding protein	1.2E-06	1.19	0.04
K00939	adenylate kinase	2.7E-02	1.19	0.01
K01951	GMP synthase (glutamine-hydrolysing)	1.6E-04	1.13	0.03
K03070	preprotein translocase subunit SecA	5.0E-15	-1.14	0.02
K01869	leucyl-tRNA synthetase	8.3E-04	-1.18	0.06
K06959	protein Tex	1.7E-11	-1.19	0.04
K07568	S-adenosylmethionine:tRNA ribosyltransferase-isomerase	4.2E-10	-1.20	0.03
K03572	DNA mismatch repair protein MutL	1.3E-05	-1.27	0.07
K02346	DNA polymerase IV	1.0E-02	-1.27	0.04
K01928	UDP-N-acetylmuramoyl-L-alanyl-D-glutamate--2,6-diaminopimelate ligase	1.4E-15	-1.34	0.03
K01662	1-deoxy-D-xylulose-5-phosphate synthase	1.8E-21	-1.34	0.06
K06187	recombination protein RecR	6.5E-20	-1.36	0.02
K06379	stage II sporulation protein AB (anti-sigma F factor)	2.8E-05	-1.37	0.02
K01963	acetyl-CoA carboxylase carboxyl transferase subunit beta	5.2E-09	-1.47	0.11
K03575	A/G-specific adenine glycosylase	4.0E-06	-1.51	0.12
K01950	NAD+ synthase (glutamine-hydrolysing)	1.4E-07	-1.51	0.15
K09903	uridylate kinase	2.1E-16	-1.51	0.12
K00560	thymidylate synthase	2.0E-03	-1.53	0.17
K00638	chloramphenicol O-acetyltransferase type B	2.5E-10	-1.58	0.15
K01449	N-acetylmuramoyl-L-alanine amidase	8.2E-05	-1.66	0.16
K00100	butanol dehydrogenase	9.6E-23	-1.70	0.08
K03402	transcriptional regulator of arginine metabolism	5.2E-11	-1.71	0.19
K00133	aspartate-semialdehyde dehydrogenase	3.3E-13	-1.71	0.15
K07566	L-threonylcarbamoyladenylate synthase	2.6E-19	-1.74	0.11
K01537	Ca2+-transporting ATPase	4.2E-22	-1.75	0.16
K03630	DNA repair protein RadC	4.7E-03	-1.84	0.33
K04020	phosphotransacetylase	2.7E-03	-2.25	0.27
K07533	foldase protein PrsA	1.3E-02	-2.33	0.59
K18673	beta-glucoside kinase	4.6E-23	-2.50	0.50
K13643	Rrf2 family transcriptional regulator, iron-sulfur cluster assembly transcription factor	6.0E-06	-2.50	0.54
K00680	[no name]	1.9E-09	-2.58	0.42
K06295	spore germination protein KA	4.0E-16	-2.67	0.19
K04019	ethanolamine utilization protein EutA	1.1E-03	-2.78	1.36
K08161	MFS transporter, DHA1 family, multidrug resistance protein	3.5E-08	-2.81	0.65
K02793	PTS system, mannose-specific IIA component	3.0E-11	-2.83	0.75
K03565	regulatory protein	7.6E-16	-2.94	0.29
K01466	allantoinase	4.0E-18	-3.00	0.69
K11737	D-serine/D-alanine/glycine transporter	2.0E-18	-3.03	0.29

KEGG number	function	adjusted *p*-value	mean fold-change (CC/F)	SEM
K18197	rhamnogalacturonan endolyase	1.6E-20	-3.06	0.36
K18198	rhamnogalacturonan exolyase	6.1E-04	-3.18	1.22
K18118	succinyl-CoA:acetate CoA-transferase	1.0E-23	-3.25	1.01
K05837	rod shape determining protein RodA	7.9E-18	-3.28	1.06
K07088	uncharacterized protein	4.5E-26	-4.00	0.87
K09698	nondiscriminating glutamyl-tRNA synthetase	2.1E-02	-4.09	1.99
K07238	zinc transporter, ZIP family	9.2E-27	-4.19	0.96
K01879	glycyl-tRNA synthetase beta chain	2.7E-15	-4.30	1.90
K03549	KUP system potassium uptake protein	9.9E-26	-4.39	0.56
K01281	X-Pro dipeptidyl-peptidase	4.7E-25	-4.69	1.68
K01220	6-phospho-beta-galactosidase	2.2E-24	-4.94	2.82
K01667	tryptophanase	1.7E-14	-5.54	3.75
K19005	lipoteichoic acid synthase	2.2E-25	-5.67	2.42
K00024	malate dehydrogenase	1.8E-27	-6.17	3.44
K12267	peptide methionine sulfoxide reductase msrA/msrB	8.2E-28	-7.33	3.10

Table S4. Bacteroidetes function with significantly differential normalized abundance between cecal and fecal metagenome.

KEGG number	function	adjusted *p*-value	mean fold-change (CC/F)	SEM
K07024	uncharacterized protein	2.0E-02	1.97	0.26
K06871	uncharacterized protein	2.0E-05	1.19	0.03
K00790	UDP-N-acetylglucosamine 1-carboxyvinyltransferase	8.4E-05	1.16	0.03
K03977	GTPase	7.3E-03	1.14	0.03
K05349	beta-glucosidase	4.8E-02	-1.13	0.01
K00262	glutamate dehydrogenase (NADP+)	7.2E-06	-1.14	0.02
K07478	putative ATPase	1.7E-03	-1.21	0.03
K01736	chorismate synthase	7.5E-04	-1.22	0.03
K02469	DNA gyrase subunit A	4.3E-06	-1.33	0.06
K08138	MFS transporter, SP family, xylose:H+ symportor	1.1E-03	-1.38	0.16
K11754	dihydrofolate synthase / folylpolyglutamate synthase	3.6E-03	-1.40	0.06
K01685	altronate hydrolase	1.4E-02	-1.43	0.10
K02316	DNA primase	5.0E-04	-1.52	0.19
K01754	threonine dehydratase	3.2E-04	-1.75	0.13
K03606	putative colanic acid biosysnthesis UDP-glucose lipid carrier transferase	1.5E-06	-1.88	0.14
K15915	undecaprenyl phosphate N,N'-diacetylbacillosamine 1-phosphate transferase	5.3E-05	-2.00	0.29
K00991	2-C-methyl-D-erythritol 4-phosphate cytidylyltransferase	2.5E-03	-2.23	0.15
K04020	phosphotransacetylase	1.2E-05	-2.30	0.15
K00567	methylated-DNA-[protein]-cysteine S-methyltransferase	1.0E-02	-2.31	0.44
K15726	cobalt-zinc-cadmium resistance protein CzcA	2.1E-04	-3.00	1.15
K00603	glutamate formiminotransferase	2.7E-02	-3.18	1.08

Table S5. Differential pathway-phylum combinations between cecal and fecal metagenome.

pathway	phylum	adjusted *p*-value	mean fold-change (CC/F)	SEM
dTDP-4-acetamido-4,6-dideoxygalactose biosynthesis	Firmicutes	2.1E-45	7.93	4.98
xyloglucan degradation	Firmicutes	5.0E-24	6.75	4.54
2-dehydro-3-deoxy-D-gluconate degradation	Firmicutes	8.6E-13	5.91	3.95
sulfite reduction	Firmicutes	1.3E-55	5.67	2.24
xylan degradation	Firmicutes	7.7E-12	5.54	3.37
3',5'-cyclic di-GMP biosynthesis	Firmicutes	1.8E-12	5.28	3.62
S-adenosylmethioninamine biosynthesis	Firmicutes	5.7E-58	5.25	1.38
peptidoglycan biosynthesis	Fusobacteria	7.6E-54	5.17	2.95
3-phenylpropanoate degradation	Firmicutes	1.9E-63	5.17	0.44
D-glycero-D-manno-heptose 7-phosphate biosynthesis	Firmicutes	1.7E-27	4.73	2.47
trehalose degradation	Firmicutes	6.1E-46	4.60	2.46
protein glycosylation	Firmicutes	2.7E-17	4.35	1.96
hydrogen sulfide biosynthesis	Firmicutes	4.8E-39	4.25	1.25
sulfatase oxidation	Firmicutes	3.6E-59	3.99	0.70
Vi-antigen biosynthesis	Firmicutes	1.2E-41	3.71	0.76
purine nucleoside salvage	Firmicutes	1.6E-35	3.57	1.00
phospholipid metabolism	Firmicutes	4.9E-18	3.52	1.22
L-arabinan degradation	Firmicutes	5.5E-61	3.49	0.40
riboflavin biosynthesis	Firmicutes	1.5E-51	3.43	0.60
L-lysine biosynthesis via AAA pathway	Firmicutes	7.5E-45	3.35	0.83
tetrahydrofolate biosynthesis	Firmicutes	4.7E-05	3.28	1.06
polypeptide chain elongation	Firmicutes	2.1E-18	3.27	0.85
7,8-dihydroneopterin triphosphate biosynthesis	Firmicutes	7.5E-07	3.18	1.14
glycerol degradation	Firmicutes	5.0E-38	3.06	0.75
poly(ribitol phosphate) teichoic acid biosynthesis	Firmicutes	1.6E-34	2.99	0.64
pectin degradation	Firmicutes	3.8E-48	2.93	0.52
propanoyl-CoA degradation	Firmicutes	4.1E-32	2.92	0.80
butanoate metabolism	Firmicutes	8.0E-63	2.90	0.20
nicotine degradation	Firmicutes	6.0E-22	2.85	0.78
L-serine biosynthesis	Firmicutes	1.4E-61	2.83	0.25
dimethylallyl diphosphate biosynthesis	Firmicutes	2.4E-07	2.79	0.69
alkane degradation	Firmicutes	5.1E-35	2.75	0.18
cellulose degradation	Firmicutes	1.3E-40	2.67	0.53
L-arginine biosynthesis	Spirochaetes	1.6E-37	2.67	0.44
hypoxanthine degradation	Firmicutes	3.6E-42	2.64	0.51
L-tryptophan biosynthesis	Firmicutes	9.7E-26	2.61	0.60
L-histidine biosynthesis	Firmicutes	9.7E-21	2.60	0.56
glycogen biosynthesis	Firmicutes	1.4E-07	2.55	0.59
L-threonine degradation via propanoate pathway	Firmicutes	4.5E-33	2.54	0.51
XMP biosynthesis via de novo pathway	Firmicutes	1.4E-32	2.49	0.48
L-arginine degradation via ADI pathway	Firmicutes	5.7E-51	2.47	0.34
(R)-mevalonate biosynthesis	Firmicutes	6.0E-28	2.43	0.50

pathway	phylum	adjusted *p*-value	mean fold-change (CC/F)	SEM
L-leucine biosynthesis	Firmicutes	9.0E-60	2.42	0.21
2-deoxy-D-ribose 1-phosphate degradation	Firmicutes	9.1E-23	2.39	0.49
L-threonine biosynthesis	Firmicutes	2.9E-53	2.38	0.19
L-isoleucine biosynthesis	Firmicutes	5.2E-37	2.34	0.37
lipoprotein biosynthesis (signal peptide cleavage)	Firmicutes	3.9E-15	2.34	0.58
tetrahydrofolate interconversion	Firmicutes	9.3E-03	2.32	0.56
dUMP biosynthesis	Firmicutes	8.9E-57	2.30	0.16
GMP biosynthesis	Firmicutes	4.4E-02	2.27	0.51
fatty acid beta-oxidation	Firmicutes	1.9E-24	2.22	0.48
phosphatidylglycerol biosynthesis	Firmicutes	6.6E-09	2.21	0.47
pentose and glucuronate interconversion	Firmicutes	1.4E-58	2.19	0.18
phenylacetate degradation	Firmicutes	1.0E-48	2.18	0.27
1,2-propanediol degradation	Firmicutes	7.4E-29	2.17	0.37
L-glutamate degradation via hydroxyglutarate pathway	Firmicutes	1.9E-13	2.16	0.45
myo-inositol degradation into acetyl-CoA	Firmicutes	1.6E-38	2.11	0.33
novobiocin biosynthesis	Firmicutes	2.4E-02	2.11	0.60
L-methionine biosynthesis via de novo pathway	Firmicutes	1.6E-11	2.10	0.40
IMP biosynthesis via de novo pathway	Firmicutes	1.5E-19	2.07	0.39
L-cysteine biosynthesis	Firmicutes	3.3E-62	2.07	0.04
nicotinate degradation	Firmicutes	1.0E-42	2.04	0.23
isopentenyl diphosphate biosynthesis via DXP pathway	Firmicutes	1.9E-14	2.01	0.36
5-phospho-alpha-D-ribose 1-diphosphate biosynthesis	Firmicutes	3.8E-06	1.96	0.38
chorismate biosynthesis	Firmicutes	2.2E-03	1.95	0.40
teichuronic acid biosynthesis	Firmicutes	8.9E-44	1.92	0.21
L-rhamnose degradation	Firmicutes	3.1E-30	1.80	0.20
(R)-pantothenate biosynthesis	Firmicutes	1.1E-52	1.76	0.09
tetrahydrofolylpolyglutamate biosynthesis	Firmicutes	4.1E-14	1.74	0.15
gluconeogenesis	Firmicutes	4.9E-10	1.69	0.24
Calvin cycle	Firmicutes	6.4E-11	1.64	0.18
lipopolysaccharide biosynthesis	Bacteroidetes	3.2E-03	-1.55	0.23
L-glutamate biosynthesis via GLT pathway	Bacteroidetes	4.6E-03	-1.64	0.34
protein glycosylation	Bacteroidetes	4.3E-07	-1.70	0.26
protoporphyrin-IX biosynthesis	Bacteroidetes	3.5E-09	-1.70	0.29
slime polysaccharide biosynthesis	Bacteroidetes	8.9E-14	-1.72	0.24
L-histidine biosynthesis	Proteobacteria	2.2E-06	-1.74	0.38
L-idonate degradation	Bacteroidetes	8.6E-19	-1.76	0.20
lipoprotein biosynthesis (signal peptide cleavage)	Bacteroidetes	1.6E-36	-1.76	0.16
4-amino-4-deoxy-alpha-L-arabinose undecaprenyl phosphate biosynthesis	Bacteroidetes	3.3E-02	-1.81	0.33
iron-sulfur cluster biosynthesis	Synergistetes	1.5E-16	-1.83	0.33
tetrahydrofolate interconversion	Euryarchaeota	1.5E-16	-1.83	0.33
L-proline biosynthesis	Bacteroidetes	2.1E-28	-1.86	0.45
capsule polysaccharide biosynthesis	Bacteroidetes	5.2E-55	-1.90	0.12

pathway	phylum	adjusted *p*-value	mean fold-change (CC/F)	SEM
xylan degradation	Bacteroidetes	2.3E-08	-1.93	0.38
N-acetylneuraminate degradation	Bacteroidetes	4.4E-04	-1.94	0.40
1-deoxy-D-xylulose 5-phosphate biosynthesis	Fusobacteria	2.9E-04	-2.03	0.32
1-deoxy-D-xylulose 5-phosphate biosynthesis	Bacteroidetes	7.6E-08	-2.06	0.42
L-isoleucine biosynthesis	Cyanobacteria	1.8E-02	-2.11	0.39
glycerol degradation via glycerol kinase pathway	Tenericutes	4.8E-34	-2.17	0.67
IMP biosynthesis via de novo pathway	Actinobacteria	3.1E-43	-2.23	0.41
oxalate degradation	Actinobacteria	4.8E-47	-2.24	0.31
alginate biosynthesis	Proteobacteria	8.6E-15	-2.25	0.38
L-methionine biosynthesis via de novo pathway	Bacteroidetes	7.8E-50	-2.31	0.28
starch degradation	Firmicutes	3.6E-31	-2.39	0.20
L-cysteine biosynthesis	Actinobacteria	6.4E-17	-2.39	0.20
peptidoglycan recycling	Bacteroidetes	7.1E-25	-2.52	0.39
galactose metabolism	Actinobacteria	1.5E-39	-2.56	0.13
L-lysine biosynthesis via DAP pathway	Spirochaetes	8.9E-30	-2.83	1.09
coenzyme A biosynthesis	Proteobacteria	6.6E-03	-2.94	1.06
S-adenosyl-L-methionine biosynthesis	Actinobacteria	4.4E-40	-2.99	1.13
UMP biosynthesis via de novo pathway	Actinobacteria	2.1E-50	-3.02	0.26
lactose degradation	Firmicutes	3.5E-23	-3.17	1.48
colanic acid biosynthesis	Bacteroidetes	1.3E-23	-3.26	0.83
slime polysaccharide biosynthesis	Proteobacteria	2.5E-10	-3.38	1.61
NAD(+) biosynthesis	Actinobacteria	2.0E-54	-3.47	0.29
ethanolamine degradation	Bacteroidetes	4.1E-52	-3.55	0.71
chorismate biosynthesis	Actinobacteria	1.2E-04	-3.81	2.00
L-glutamate biosynthesis via GLT pathway	Actinobacteria	1.1E-05	-5.17	3.35
L-methionine biosynthesis via de novo pathway	Actinobacteria	2.9E-49	-7.03	3.81
