# Supplementary material for: Metaproteogenomics Reveals Taxonomic and Functional Changes between Cecal and Fecal Microbiota in Mouse
Source: Front Microbiol. 2017 Mar 14;8:391. doi: 10.3389/fmicb.2017.00391 (PMC5348496; doi:10.3389/fmicb.2017.00391)
Supplement: Supplementary file 3 [file Image_1.pdf]

Supplementary Figures

Metaproteogenomics reveals taxonomic and functional changes between cecal and fecal microbiota in mouse

Alessandro Tanca, Valeria Manghina, Cristina Fraumene, Antonio Palomba, Marcello Abbondio, Massimo Deligios, Michael Silverman, and Sergio Uzzau*

*** Correspondence:** Sergio Uzzau: uzzau@portocontericerche.it

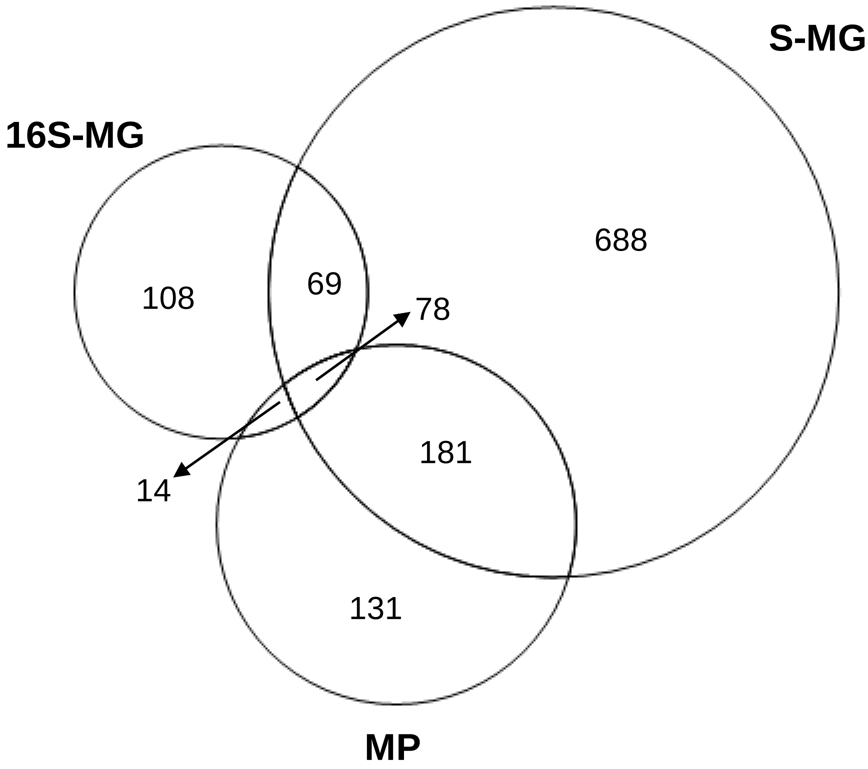

Figure S1. Overlap among genera detected by 16S-MG, S-MG and MP (irrespective of the sample of origin).

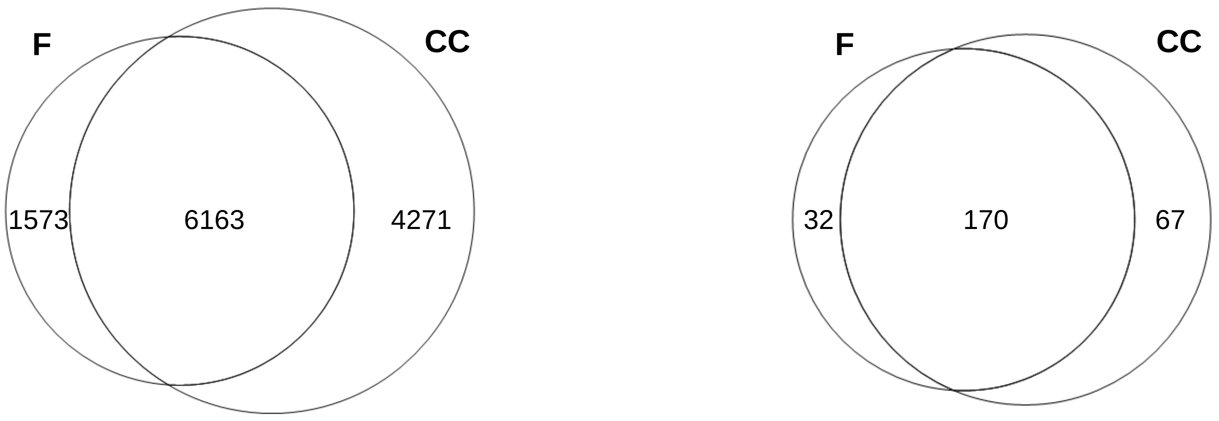

Figure S2. Overlap among OTUs (left) and genera (right) detected by 16S-MG in cecal contents (CC) and feces (F).

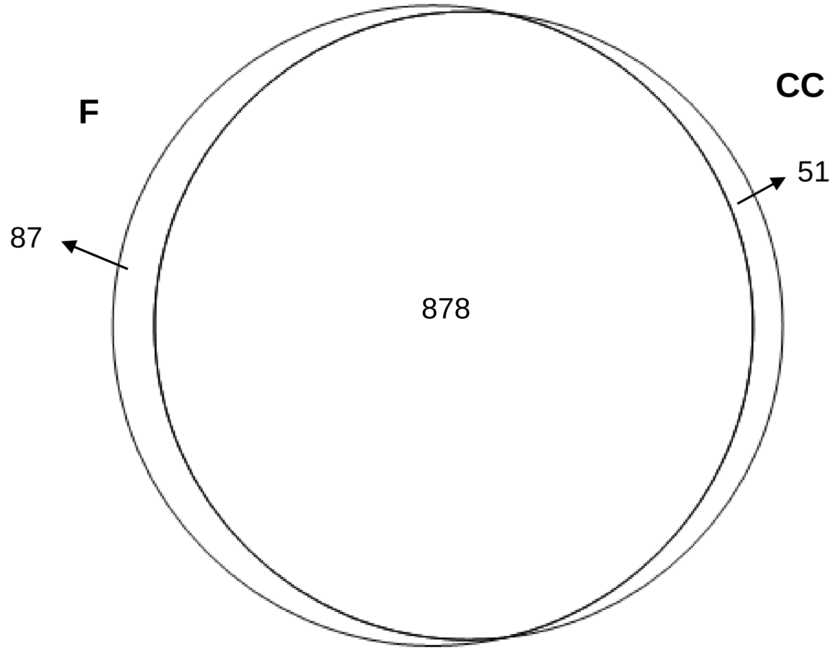

Figure S3. Overlap among genera detected by S-MG in cecal contents (CC) and feces (F).

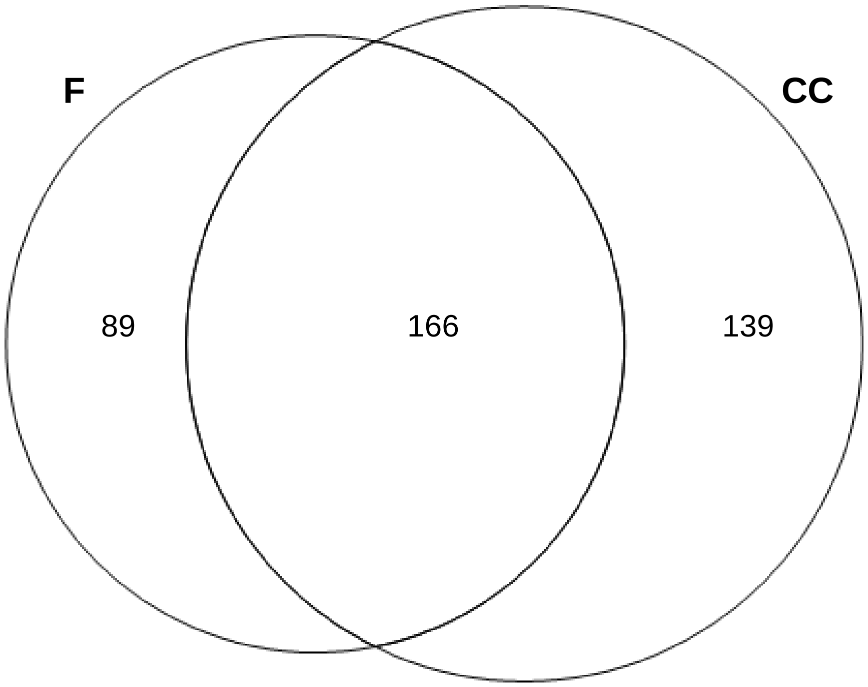

Figure S4. Overlap among genera detected by MP in cecal contents (CC) and feces (F).

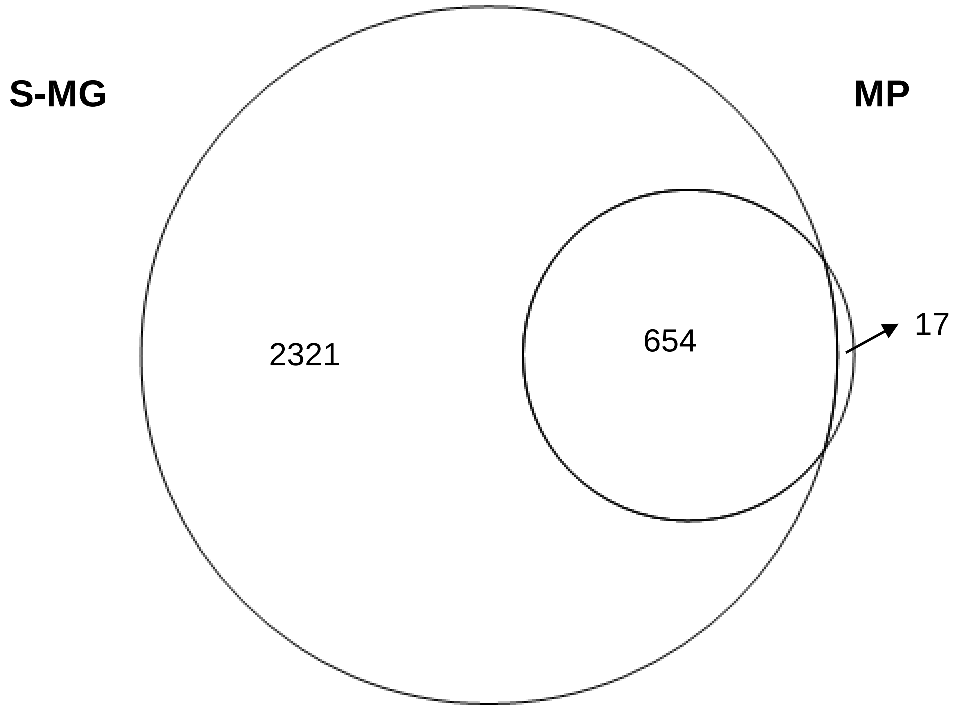

Figure S5. Overlap among functions detected by S-MG and MP (irrespective of the sample of origin).

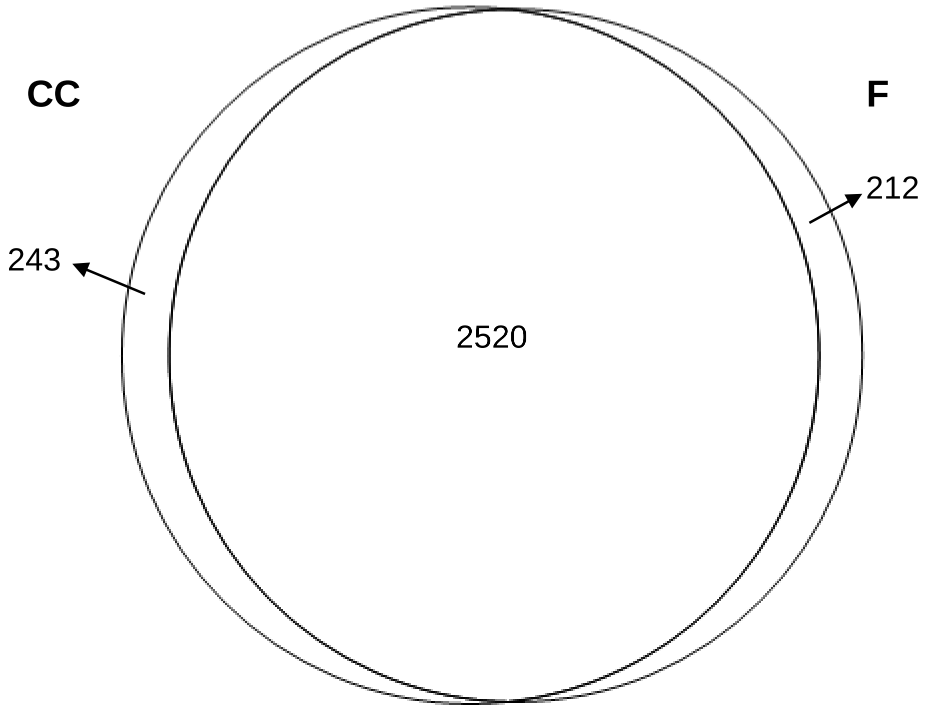

Figure S6. Overlap among functions detected by S-MG in cecal contents (CC) and feces (F).

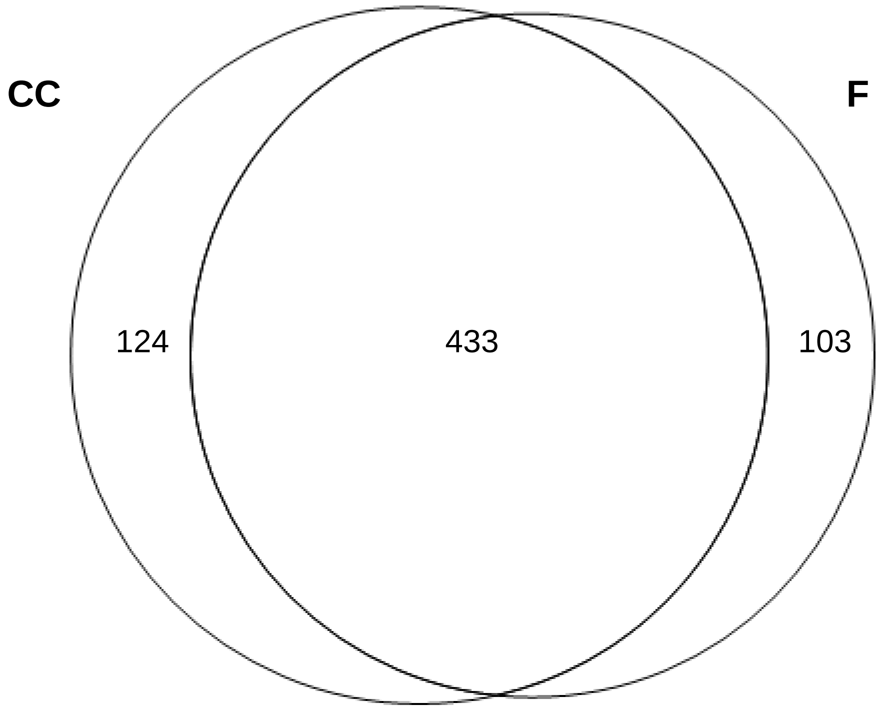

Figure S7. Overlap among functions detected by MP in cecal contents (CC) and feces (F).

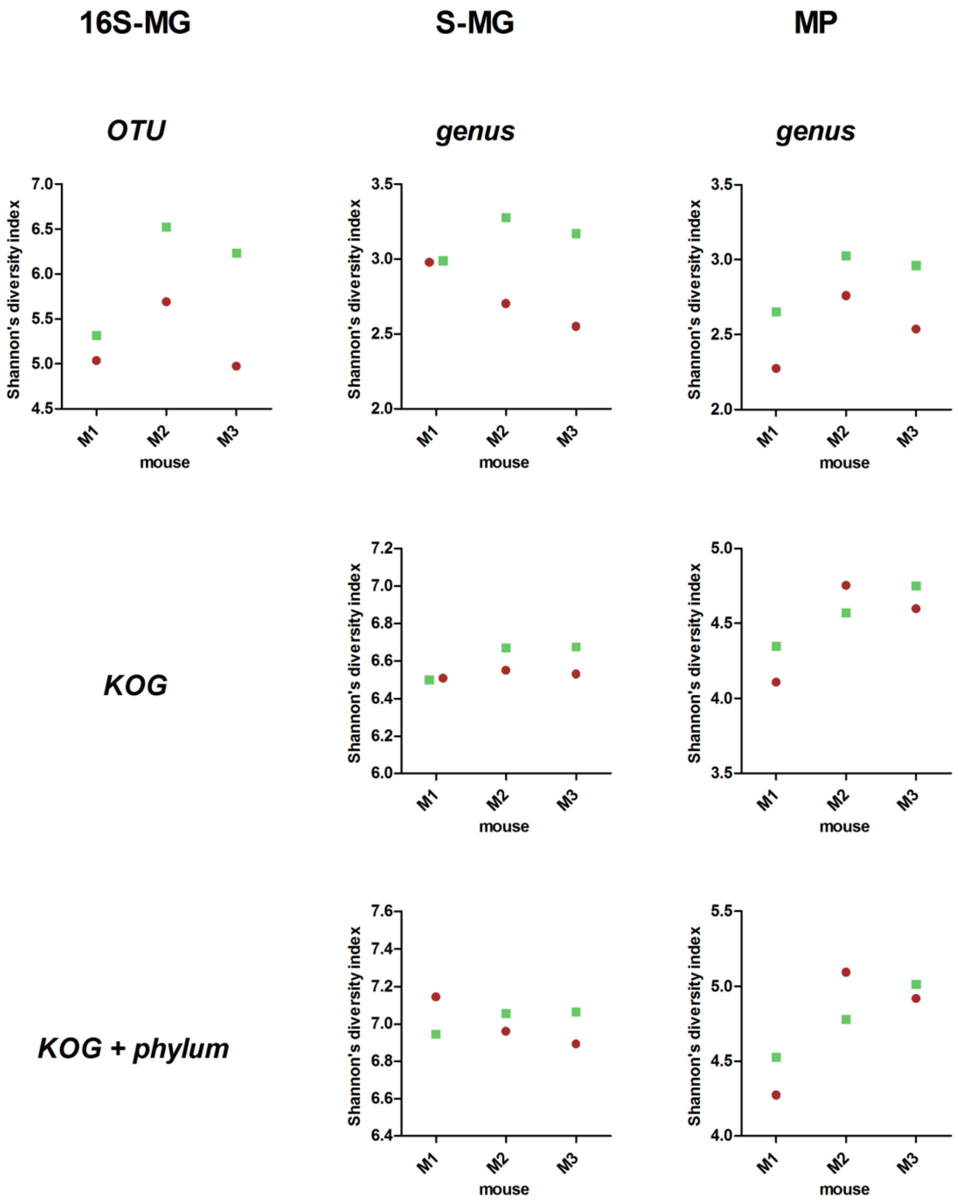

Figure S8. Alpha-diversity at taxonomic, functional and combined functional/taxonomic level in caecal contents (green) and feces (brown). Shannon's diversity index was calculated based on 16S-MG (left), S-MG (center) and MP (right) data.

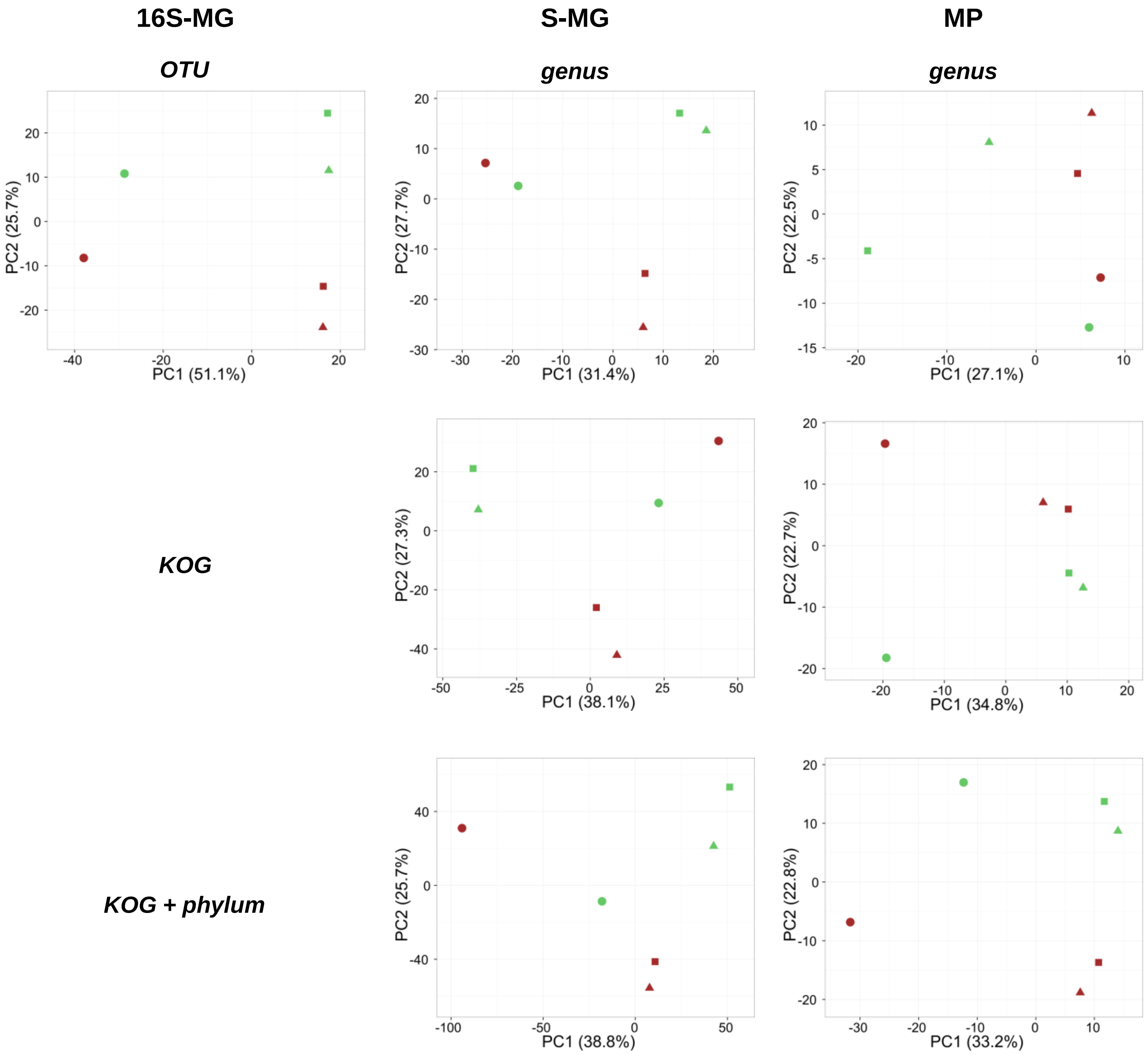

Figure S9. Beta-diversity at taxonomic, functional and combined functional/taxonomic level among caecal content (green) and fecal (brown) samples. Each dot represents a different sample, with the same shape indicating the same mouse. Principal component analysis was carried out on 16S-MG (left), S-MG (center) and MP (right) data.

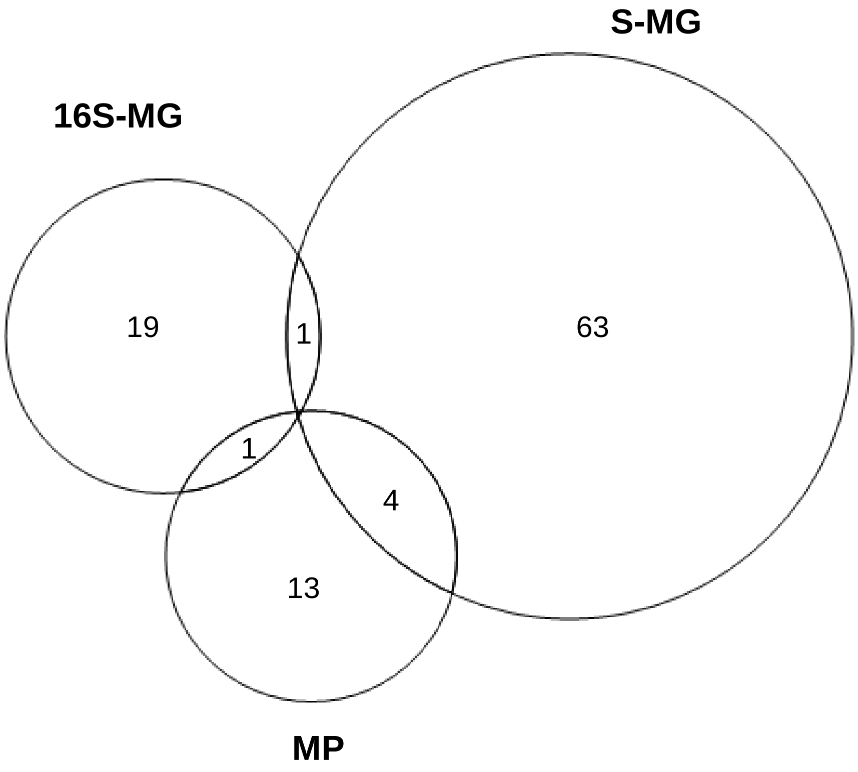

Figure S10. Overlap among genera detected by 16S-MG, S-MG and MP with differential abundance between cecal contents and feces.

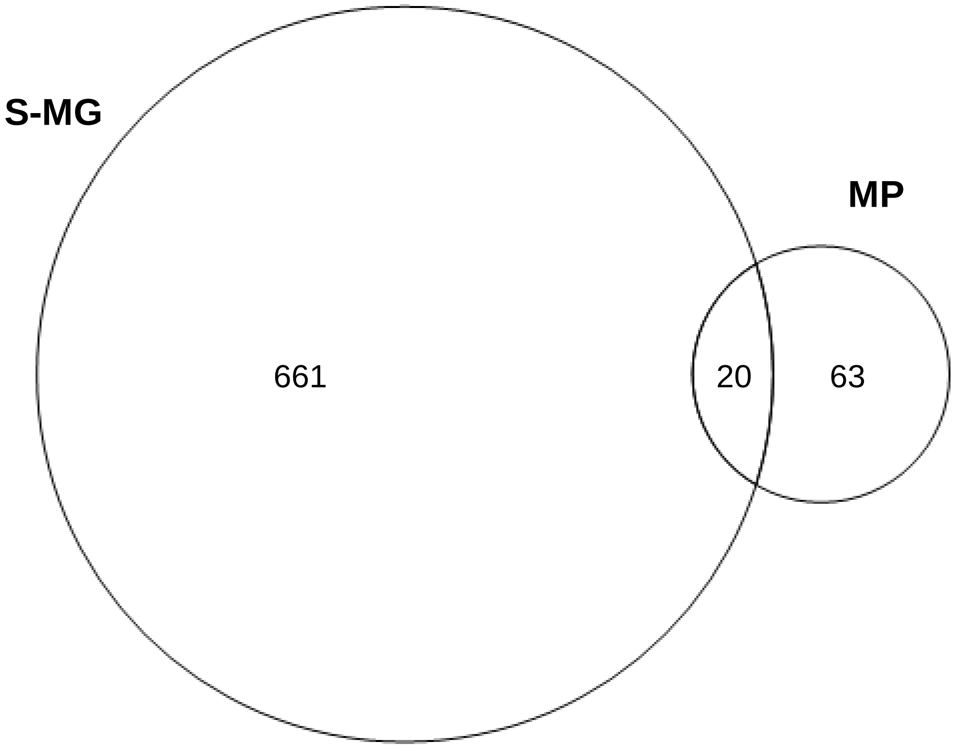

Figure S11. Overlap among function-phylum combinations detected by S-MG and MP with differential abundance between cecal contents and feces.
